# Triboelectric Nanogenerators in Military: Recent Progress and Critical Challenges

**DOI:** 10.1007/s40820-026-02221-9

**Published:** 2026-06-04

**Authors:** Changcheng Bao, Xiaoxia Ma, Min He, Li Yang, Yingting Wang

**Affiliations:** 1 School of Mechatronical Engineering, Beijing Institute of Technology, Beijing, 100081 People’s Republic of China; 2 State Key Laboratory of Explosion Science and Safety Protection, Beijing Institute of Technology, Beijing, 100081 People’s Republic of China; 3 The Institute of Precision Machinery and Smart Structure, College of Engineering, Zhejiang Normal University, Yingbin Street 688, Jinhua, 321004 People’s Republic of China

**Keywords:** Triboelectric nanogenerator (TENG), Military, Self-powered sensing, Energy harvesting

## Abstract

The rapid evolution of modern warfare toward distributed, intelligent, and global stealth has posed severe energy supply and information perception bottlenecks for combat nodes. Traditional energy supply strategies are unable to meet the strategic needs of long-range stealth operations and extreme environmental survival. Triboelectric nanogenerators provide a disruptive path for constructing self-sustaining military microsystems with high-efficiency and zero-power-sensing characteristics. This review establishes a comprehensive framework that systematically evaluates recent technological advances across an escalating hierarchy, encompassing individual soldier combat platforms, unmanned combat systems, strategic aerospace equipment, and special tactical scenarios. Crucially, moving beyond generic laboratory demonstrations, this review adopts a rigorous defense engineering perspective by conducting an integrated failure mode analysis to evaluate device degradation under severe battlefield stressors. Furthermore, it critically analyzes the primary engineering bottlenecks currently hindering military adoption, such as inherent impedance mismatch and scalable manufacturing barriers, and proposes targeted mitigation strategies. To systematically validate these solutions, we explicitly propose a concise evaluation framework strictly aligned with established military testing protocols. Ultimately, this review outlines several forward-looking strategic priorities, thereby constructing a highly actionable roadmap to accelerate the practical deployment of self-driven intelligent technologies.

## Highlights

- Recent advances of triboelectric nanogenerators are systematically reviewed across multiscale military platforms, including individual soldier systems, unmanned combat platforms, strategic equipment, and special tactical scenarios.
- Core advantages of triboelectric nanogenerators in structural-functional integration, extreme environment robustness, and synergy of self-powered energy supply and sensing for military demands are highlighted.
- Critical engineering bottlenecks are analyzed, and forward-looking directions (e.g., physical layer encryption, self-powered Internet of Military Things) are proposed to accelerate practical deployment.

## Graphical abstract

**Figure:**
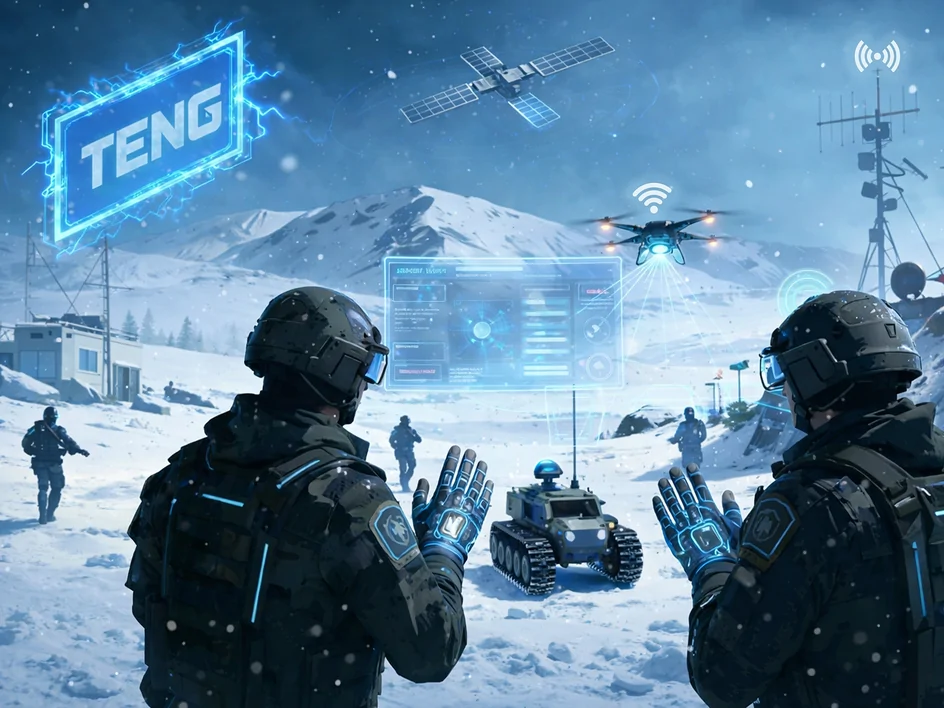

## Introduction

Modern warfare is rapidly advancing into a new phase of joint operations characterized by informatization and intelligence [1, 2]. The large-scale deployment of individual soldier smart terminals, long-endurance unmanned combat platforms, high-precision guided weapons, and distributed battlefield sensor networks has posed revolutionary demands on energy supply systems and all-domain state monitoring technologies [3–6]. However, the inherent flaws of traditional military energy and sensing architectures are becoming increasingly prominent and have become strategic bottlenecks that hinder the enhancement of the combat effectiveness of new-generation equipment [7–11]. In terms of energy supply, wired power supply models are constrained by battlefield terrain and mobility requirements, making them difficult to adapt to the flexible deployment of swarm drones or distributed nodes in the wild [12–16]. Meanwhile, chemical battery energy storage faces issues such as performance degradation at low temperatures, short-endurance cycles, maintenance difficulties, and high logistical supply pressure [17–20]. Especially in extreme environments such as the polar regions, deep oceans, or areas contaminated by nuclear, biological, and chemical weapons, frequent battery replacement and charging operations not only put a huge strain on logistical supply lines but also easily reveal combat intentions, significantly reducing the battlefield survivability of special forces and stealth equipment [21–24]. In terms of state monitoring, existing sensing systems rely heavily on external energy supply and lack adaptability to complex battlefield environments [25–27]. High-temperature differences can easily lead to sensing accuracy drift, strong electromagnetic interference can cause signal distortion, and severe vibration may damage device structures, making it difficult to achieve real-time monitoring of individual soldier equipment, unmanned platforms, and large combat vehicles throughout their entire lifecycle [28–32]. Furthermore, lightweight and concealment are core principles in modern equipment design [33–37]. Traditional energy supply and sensing modules are bulky and energy-intensive, making them difficult to integrate into micro-/nanoscale military equipment, miniaturized, and flexible military equipment, further limiting the expansion of tactical application scenarios [38–40]. Therefore, the key to breaking this deadlock lies in finding a disruptive technological solution that can directly extract energy from the battlefield environment and achieve self-driving, all-weather perception [41, 42].

To address these challenges, the emergence of triboelectric nanogenerators (TENGs) offers a novel technological path for constructing self-sustaining military microsystems for the future [43–47]. As an emerging energy technology based on Maxwell’s displacement current theory, TENGs can efficiently convert the widely existing, low-frequency, and disordered mechanical energy in the environment into high-quality electrical energy through the coupling effect of contact electrification and electrostatic induction [48–54]. Compared with traditional electromagnetic generators (EMG) or piezoelectric sensors, TENG possesses unparalleled unique advantages: Firstly, it has a wide range of energy sources and can effectively collect ubiquitous “waste energy” on the battlefield, including bio-mechanical energy generated by soldiers’ tactical movements, vibration energy during equipment operation, natural wind energy, wave energy, and even acoustic energy, fundamentally endowing equipment with energy autonomy [55–58]. Secondly, it has an extremely rich selection of materials [59]. Based on micro-/nanomaterial design and regulation strategies, almost all polymers, metals, and even biological materials can be used to construct TENG, providing infinite possibilities for adapting to complex physical and chemical environments on the battlefield [60–62]. Thirdly, it possesses natural sensing characteristics [63]. The electrical output signal of TENG has a precise linear or nonlinear correspondence with external mechanical stimuli, making it possible to function as a high-sensitivity active sensor without an external power source, enabling real-time sensing of the battlefield situation [64, 65]. These characteristics make TENG an ideal technological cornerstone for building future self-sustaining and intelligent military microsystems.

More importantly, the inherent compatibility between TENG and military requirements is driving equipment design to make a deep leap toward structural and functional integration [66]. This paradigm shift is reflected in the following dimensions: firstly, zero burden integration, where TENG can directly utilize existing textiles, packaging layers, or structural components to construct passive protective equipment into active energy-sensing nodes, achieving functional doubling without increasing individual load capacity [67–69]; secondly, there is intrinsic safety, as the solid-state structure based on polymer dielectrics completely avoids the risk of explosion of traditional chemical power sources under shrapnel puncture or high temperature, significantly improving the battlefield survivability of equipment [70, 71]; finally, there is full-spectrum stealth adaptation, whose non-metallic substrate naturally has low radar cross section and low infrared radiation characteristics, and is easy to achieve optical transparency and acoustic impedance matching, perfectly meeting the stringent requirements of modern warfare for global stealth and silent reconnaissance [72, 73]. It can be said that TENG is not only an energy technology but also a strategic empowerment technology that can comprehensively reshape the battlefield and enhance the comprehensive combat capabilities of individual soldiers and platforms from the bottom up.

Given the immense potential of TENGs in reshaping defense energy and sensing systems, this review systematically reviews the latest advancements and evolutionary trends of TENGs in the modern military field. Although several recent reviews have explored this technology, the majority of these works primarily focus on fundamental mechanisms, general self-powered sensors, or civilian wearable electronics. When military applications are mentioned in existing literature, they are frequently treated as brief extensions of civilian technologies, lacking a comprehensive analysis of the rigorous demands inherent in actual combat environments. To firmly position this review within the existing literature and bridge this critical gap, this review introduces a highly cross-disciplinary framework and fundamentally differentiates itself by adopting a strict engineering application perspective tailored to military standards. This review strategically reorients the focus toward explicit tactical engineering requirements by systematically organizing the applications around escalating military platform scales. By adopting this platform-centric approach, this review uniquely maps the intrinsic physical attributes of these advanced materials directly to the most critical battlefield bottlenecks. This strategic mapping emphasizes how self-powered architectures fundamentally resolve the vulnerabilities of traditional power supplies by ensuring electromagnetic stealth compatibility, facilitating unhackable physical layer information security, and guaranteeing operational robustness under extreme combat conditions. Furthermore, this work provides a dialectical analysis of the severe engineering bottlenecks currently hindering military adoption, thereby constructing a pragmatic roadmap that bridges the divide between basic materials science and next-generation self-powered defense architectures.

**Fig. 1:**
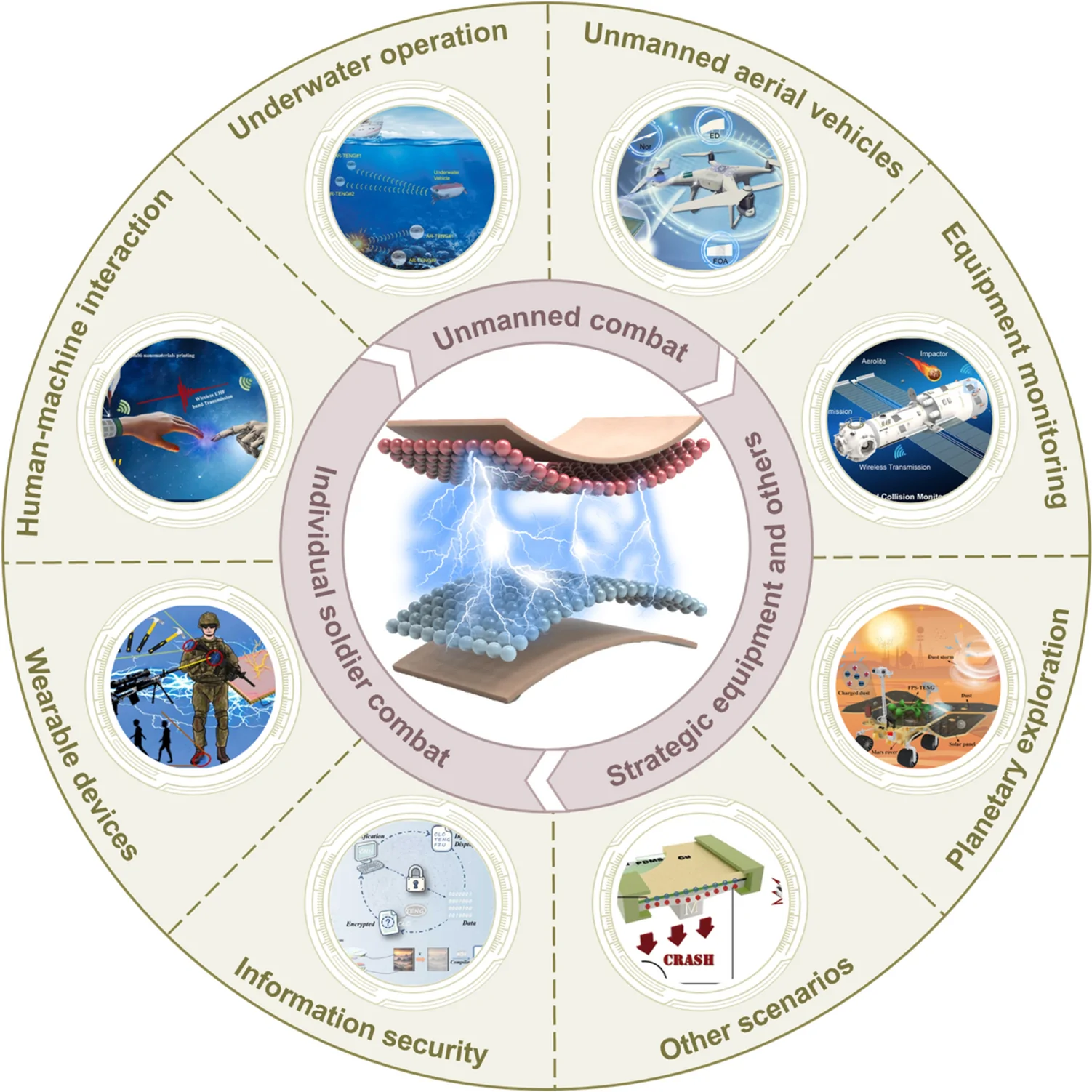
Strategic layout of TENG-enabled next-generation defense systems

The logical structure of this review is organized as follows. Chapter 2 solidifies the theoretical foundation by elucidating the working mechanisms and introducing material design strategies specifically adapted to extreme military environments. Chapter 3 serves as the core analytical section, completely restructured to categorize applications strictly by military platform type, encompassing the individual soldier combat platform, the unmanned combat platform, the strategic equipment platform, and other special tactical scenarios. Chapter 4 dialectically analyzes the critical engineering bottlenecks in moving toward practical deployment, such as inherent impedance mismatch and scalable manufacturing barriers, while proposing targeted engineering countermeasures. Finally, Chapter 5 summarizes the core viewpoints, clarifies future research priorities, and outlines the strategic blueprint for self-powered technology in future intelligent and all-domain warfare. This comprehensive framework aims to provide a systematic reference for scholars and accelerate the implementation of self-powered intelligent technologies in the modern military field. Figure 1 shows the strategic layout of the next-generation defense system with TENG functionality.

## Fundamental Principles and Working Modes of TENG

### Fundamental Principle of TENG

The engineering term triboelectrification (TE) is used to describe the general phenomenon of contact electrification (CE) [74]. Although triboelectrification has been known for 2600 years, it remains deeply puzzling to those who have devoted themselves to studying it [75, 76]. There is still controversy over whether contact electrification is caused by electron transfer, ion transfer, or material-type transfer [77–81]. This may be due to limitations in measurement techniques and the complexity of contact electrification during friction processes [82]. As a typical engineering application of the triboelectrification principle, the basic working mechanism of TENGs can be systematically explained from the more fundamental Maxwell displacement current theory [83]. The Maxwell equations unify the concepts of electric and magnetic fields and have significant importance in contemporary basic science and technology [84]. The traditional Maxwell’s fundamental equations are applicable to media with fixed boundaries and volumes, which are in a relatively static state. The source equations are [83, 85]:

1 $$\nabla \cdot{\boldsymbol{D}} = \rho_{f}$$

2 $$\nabla \cdot{\boldsymbol{B}} = 0$$

3 $$\nabla \times {\boldsymbol{E}} = - \frac{\partial }{\partial t}{\boldsymbol{B}}$$

4 $$\nabla \times {\boldsymbol{H}} = {\boldsymbol{J}}_{f} + \frac{\partial }{\partial t}{\boldsymbol{D}}$$

In the formula, ***D*** represents the electric displacement vector; ***B*** represents the magnetic induction intensity; ***E*** represents the electric field intensity; ***H*** represents the magnetic field intensity; ***J***_f_ represents the free current density.

Wang et al. explained how the output of TENG originates from Maxwell’s displacement current and added an additional term ***P***_s_ to the displacement vector ***D*** to clarify the contribution of static charges caused by contact electrification and relative motion or shape changes of the medium during mechanical excitation to its power generation [86, 87]. Therefore, the new electric displacement vector ***D*** can be written as:

5 $$\user2{D = }\varepsilon_{0} {\boldsymbol{E}} + {\boldsymbol{P}} + {\boldsymbol{P}}_{S}$$

In the formula, *ε*_0_ represents the vacuum dielectric constant. The first polarization vector ***P*** is caused by the induction effect of the external electric field, while the added term ***P***_s_ is mainly the polarization effect generated by the mechanical drive caused by surface charge and dielectric motion. Substitute Eq. (5) into Maxwell’s equations and define:

6 $${\boldsymbol{D}}^{\prime}\user2{ = }\varepsilon_{0} {\boldsymbol{E}} + {\boldsymbol{P}}$$

Therefore, Maxwell’s equations can be extended into a new system of consistent equations:

7 $$\nabla \cdot{\boldsymbol{D}}^{\prime} = \rho - \nabla \cdot{\boldsymbol{P}}_{S}$$

8 $$\nabla \cdot{\boldsymbol{B}} = 0$$

9 $$\nabla \times {\boldsymbol{E}} = - \frac{\partial }{\partial t}{\boldsymbol{B}}$$

10 $$\nabla \times {\boldsymbol{H}} = {\boldsymbol{J}} + \frac{{\partial {\boldsymbol{P}}_{S} }}{\partial t} + \frac{{\partial {\boldsymbol{D}}^{\prime}}}{\partial t}$$

According to Eq. (10), the conduction current is ***J***, and the total displacement current is:

11 $${\boldsymbol{J}}_{D} \user2{ = }\frac{{\partial {\boldsymbol{D}}^{\prime}}}{\partial t} + \frac{{\partial {\boldsymbol{P}}_{S} }}{\partial t}$$

**Fig. 2:**
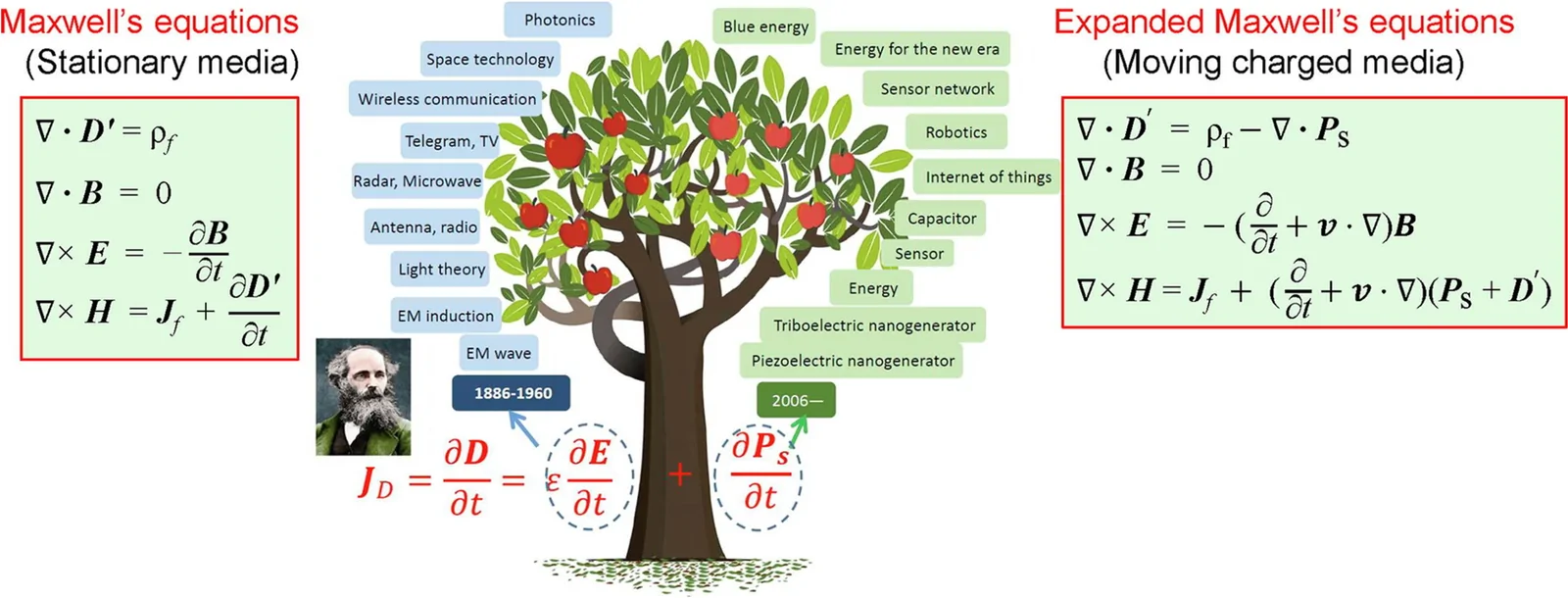
Comparison of Maxwell’s equations for stationary media and moving charged media. Reproduced with permission from Ref. [88]. Copyright 2022, Elsevier

The first term on the right side of the equation represents the displacement current caused by the variation of the electric field over time; the second term represents the current caused by changes in the boundary of the medium. These equations are the basis for deriving the output characteristics of TENG. Figure 2 shows Maxwell’s equations for a stationary medium and a moving charged medium, respectively. Based on Maxwell's displacement current theory, Wang et al. fundamentally unified the working mechanism of TENG, marking a significant expansion of classical electromagnetism in the field of energy harvesting.

The extension of Maxwell’s equations provides the specific mathematical foundation for next-generation military-oriented device design. By introducing the mechanically triggered polarization term into the displacement current equation, this theoretical framework explains why these devices inherently excel at capturing the low-frequency and highly disordered mechanical energy ubiquitous in combat environments, such as infantry tactical movements and chaotic armored-vehicle vibrations. Unlike traditional electromagnetic generators, which rely on the time derivative of magnetic flux and therefore require high-speed continuous rotation to maintain efficiency, this polarization-driven displacement current can sustain efficient energy conversion even under highly irregular and low-frequency battlefield excitations. Furthermore, the fundamental reliance on the polarization term dictates that the energy generation architecture primarily employs dielectric polymers rather than heavy metallic coils and magnets. This intrinsic material requirement, derived directly from the expanded theoretical equations, naturally endows the resulting military devices with several tactical advantages, including extreme lightweight characteristics for individual soldier payloads and inherent wave-permeable properties for radar-stealth operations. Most importantly, the ability to generate a measurable displacement current without any external power source provides the physical basis for zero-power tactical sensing. This allows distributed combat troops and autonomous unmanned systems to perceive environmental threats completely passively, thereby ensuring strict radio silence and avoiding the thermal or active electromagnetic signatures typical of conventional sensors. Therefore, this expanded theoretical foundation does not merely describe a general physical phenomenon, but directly informs the stealthy, highly conformal, and robust engineering paradigms required for multidomain military operations.

### Working Modes of TENG

**Fig. 3:**
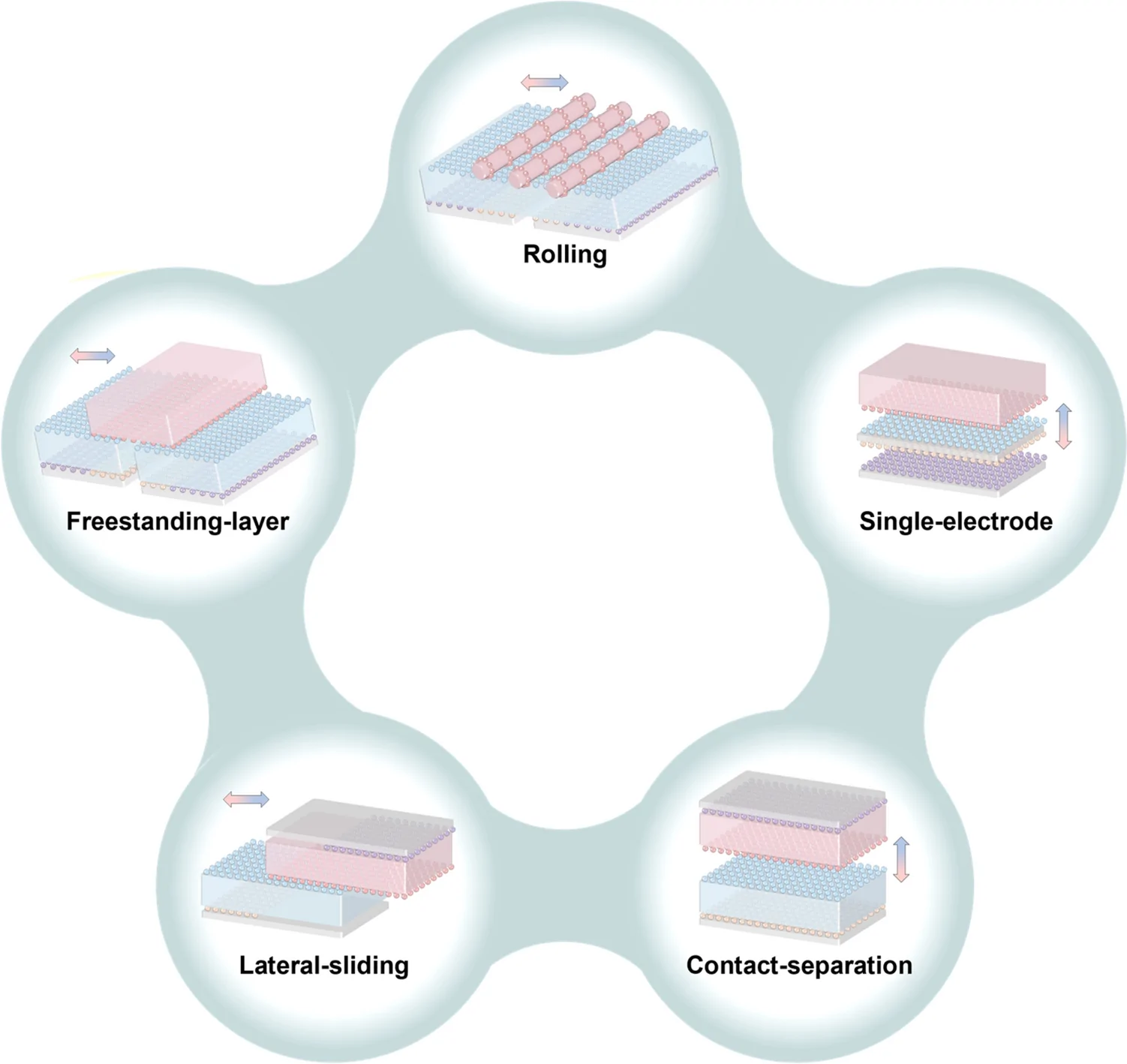
Five working modes of TENG

In response to the differences in mechanical energy forms in different application scenarios, researchers have developed four basic working modes: contact-separation, lateral-sliding, single-electrode, and freestanding-layer, as well as a rolling mode that integrates multi-mode characteristics (Fig. 3) [89]. These five modes have their own characteristics in structural design, charge transfer path, energy output characteristics, etc. They demonstrate differentiated advantages in friction loss control, output stability, and equipment integration adaptability and can well adapt to the diverse low-frequency mechanical energy capture needs in the military and aerospace fields.

#### Contact-Separation Mode

The contact-separation mode is the most basic and widely used working mode of TENG, with its core principle based on the reciprocal contact and separation between two triboelectric materials with different surface charge polarities [90, 91]. When two materials come into contact, triboelectric charges with opposite polarities are generated on the surfaces. When separated under external force, a gap is created between the two surfaces, leading to changes in the electric field distribution. This creates a potential difference between the back electrodes through electrostatic induction, driving electrons to flow in the external circuit to balance the potential. Once the gap narrows, the potential difference generated by the triboelectric charges disappears, and the induced electrons flow back. The periodic contact and separation between the two materials drive the induced electrons to flow back and forth between the two electrodes, resulting in AC output in the external circuit [92, 93]. This mode is characterized by simple structure, easy manufacturing, and high instantaneous power density, making it very suitable for collecting mechanical energy in the form of pressing, tapping, or vibration [94].

#### Lateral-Sliding Mode

The lateral-sliding mode is primarily designed for relative motion in the planar direction [95, 96]. Its working principle relies on the relative sliding of two layers of triboelectric materials that maintain contact to change the effective contact area. Under external force driving, the two triboelectric layers slide relative to each other in the horizontal direction, disrupting the charge distribution balance between the electrodes, thereby generating an electric potential difference that drives free electrons to move directionally in the external circuit, forming an induced current. When the two triboelectric layers slide in the opposite direction to restore the initial contact state, the charge distribution tends to balance again, and electrons flow in the opposite direction [97]. This process can be repeated to achieve continuous power output. The core characteristics of this mode are that the output performance is positively correlated with sliding frequency and stroke, the energy conversion efficiency is stable, the structural design is simple, the triboelectric layer wear is uniform, and it has good long-term service stability [98]. At the same time, the output power can be flexibly regulated by optimizing the triboelectric layer area and sliding path [99, 100].

#### Single-Electrode Mode

The single-electrode mode was invented to address the practical pain point of connecting wires to moving objects [101]. Its structural feature is that only one end, which is stationary, is equipped with an electrode and grounded (or referenced to a potential). The moving charged object itself serves as the other electrode. The principle behind it is that when the charged object approaches or moves away from the grounded electrode, it changes the induced charge density on the electrode through electrostatic induction, thereby driving electrons to flow between the electrode and the ground [102, 103]. The greatest value of this mode lies in granting high degrees of freedom to moving components, without any cable constraints. Therefore, it holds an irreplaceable position in the field of human–computer interaction and is widely used in touch screens, self-powered keyboards, electronic skins, as well as wireless energy harvesting and sensing for human motion or rotating tires [104].

#### Freestanding-Layer Mode

The freestanding-layer mode is an advanced improvement of the sliding mode, designed to reduce frictional loss and enhance durability [105]. Its structure comprises a pair of stationary metal electrodes and a charged moving dielectric layer (freestanding-layer), which moves or slides above the two stationary electrodes. Through electrostatic induction, the potential of the two electrodes is alternately changed, driving electrons to flow back and forth between the two stationary electrodes [106, 107]. The most notable feature of this mode is its ability to achieve non-contact power generation, thereby significantly reducing material wear, greatly extending the lifespan of the device, and achieving extremely high energy conversion efficiency [108]. This makes it highly suitable for long-term stable operation scenarios, such as non-contact speed sensors, automotive exhaust flow energy harvesting, and high-performance rotary generators [109, 110]. Furthermore, since the output performance of this mode is profoundly influenced by external excitation frequency, optimizing the matching relationship between structural parameters and mechanical motion frequency can significantly maximize low-frequency energy harvesting [111]. This frequency-dependent dynamic characteristic provides crucial theoretical guidance for designing robust devices capable of adapting to the broadband and disordered vibration environments typical of military platforms.

#### Roll Mode

Rolling mode is a composite mode formed by combining the above four modes [112, 113]. Its core relies on the rolling contact electrification effect between the sphere or ball stick and the solid friction interface to achieve electrical energy output. The specific mechanism is that when the rolling elements with different electronegativity come into contact with the fixed triboelectric layer, electron transfer occurs, and an equal amount of opposite-sign electrostatic charges are formed. The external force driving the rolling elements to roll along the interface will dynamically change the contact area. With the reasonable layout of the circular or partitioned electrodes below, it can continuously break the balance of charge distribution between the electrodes and generate a stable potential difference and induced current [114]. The outstanding advantage of this mode is that the rolling contact is a point contact and the contact position is continuously switched, which can significantly reduce friction loss and material wear, improve the long-term service stability of the device, and maintain efficient charge transfer efficiency and output performance [115]. It is suitable for scenarios involving low-frequency rolling or rotating motion, such as unmanned vehicle wheel rolling power generation, and deep-sea exploration equipment propeller shaft rotation power generation [116].

To systematically evaluate the tactical applicability of these fundamental mechanisms, it is essential to explicitly compare the five working modes across critical military engineering parameters. Although the underlying displacement current theory governs all configurations, their distinct structural designs lead to markedly different operational capabilities in actual combat environments. Therefore, this review summarizes these differences in Table 1, which evaluates each mode in terms of environmental robustness, structural integration difficulty, and inherent output characteristics. By cross-referencing these performance characteristics with military robustness requirements, engineers can more effectively identify the optimal working mode for specific tactical platforms.

**Table 1:** Systematic comparison of the five working modes across critical military engineering parameters

Working mode	Robustness	Integration difficulty	Power output characteristics	Military applications
Contact-separation	Moderate	High	High peak output under intermittent excitation	Armored vehicle vibration monitoring
Lateral-sliding	Low	Moderate	Continuous AC output under sliding motion	Sliding-part sensing
Single-electrode	High	Very low	Flexible output for freely moving targets	Smart wearables and HMI
Freestanding-layer	High	Low	High-efficiency output with low wear	Rotary sensing and aviation monitoring
Rolling	Very high	Moderate	Stable output with reduced wear	UUVs and planetary exploration

### Materials Selection and Classification for TENG

The core of material selection for TENGs lies in maximizing the surface charge density of the contact interface, which directly depends on the relative position of the paired materials in the triboelectric sequence and their intrinsic ability to gain and lose electrons [117–120]. However, the selection of TENG materials for military applications must go beyond the physical level of simply pursuing high surface charge density and instead seek a rigorous balance between electrical output and battlefield environmental survivability [121]. Although maximizing the charge density of the contact interface based on the triboelectric sequence is still the core principle, environmental adaptability often plays a decisive role in military applications [122, 123]. In the selection of negative electrode materials, polymers containing strong electron-withdrawing fluorine atoms (such as PTFE, FEP, and PVDF) have become the mainstream cornerstone for building high-performance devices due to their excellent electron-withdrawing ability [124]. However, in extreme working conditions that are difficult for ordinary polymers to withstand in aerospace and deep space exploration, polyimide (Kapton) and polyetheretherketone (PEEK) have become strategic materials for ensuring the continuous operation of devices in near Earth space or nuclear, biological, and chemical environments due to their excellent thermal stability, radiation resistance, and chemical inertness [125, 126]. Relatively speaking, polymers rich in electron-donating groups (such as nylon, silk) and even human skin are ideal positive electrode materials, while metals such as copper and aluminum are often designed as dual-functional structures that combine electrodes and triboelectric layers to simplify the system [127, 128]. In order to overcome the limitations of intrinsic material properties, surface engineering techniques such as micro-/nanostructured lithography and ion implantation have been widely adopted [129, 130]. This not only greatly improves the charge transfer efficiency but also endows the material with superhydrophobic and self-cleaning properties, ensuring the reliability of the device in complex tactical environments such as humid jungles or oceans.

At the same time, the evolution of electrode materials is driving TENG to transition from traditional rigid blocks to stealth and conformal flexible systems, which is crucial for individual equipment and covert operations in modern warfare. Although traditional gold, silver, and copper thin films have excellent conductivity, their rigidity limits seamless integration with soldiers’ wearable equipment [131]. Therefore, carbon-based nanomaterials (such as graphene and carbon nanotubes) and metal nanowires (such as silver nanowires) have become the core choices for intelligent military uniforms and soft robots due to their excellent fatigue resistance and potential electromagnetic shielding ability under large strains [132]. In order to meet the optical stealth requirements in special operations, indium tin oxide and transparent conductive materials such as ultra-thin silver films have achieved compatibility between energy harvesting and visual concealment, enabling TENG to be integrated on solar panels, helmet masks, or display terminal surfaces without obstructing the line of sight [133, 134]. In addition, for naval and diving combat scenarios, hydrogel and ionic gel plasma conductors use their modulus adjustability to solve the interface mismatch problem between rigid electrons and soft biological tissues or water media, not only realizing bio-integrated sensing, but also achieving acoustic impedance matching with water, providing an ideal material solution for underwater covert communication and bionic camouflage [135, 136]. Overall, these developments indicate that TENG material design is evolving from the isolated optimization of charge generation toward the integrated coordination of structure, function, and environment. To further translate these advanced material systems into practical military devices, researchers have increasingly adopted additive manufacturing techniques, especially three-dimensional printing, to fabricate complex, lightweight, and conformal energy-harvesting structures tailored to tactical gear [137]. These manufacturing strategies not only expand the structural versatility of TENG materials but also provide the technological basis for integrating intelligent sensing modules into complex military equipment and soldier uniforms.

## Strategic Applications of TENGs in Multiscale Military Platforms

Before delving into specific military platforms, it is crucial to clearly delineate the foundational operational paradigms of TENGs, namely, energy harvesting and self-powered sensing. Although both originate from the same Maxwell displacement current, their integration pathways in military systems are distinctly different. For energy-harvesting applications, the primary objective is to maximize the time-averaged power output. This typically requires power management circuits, such as rectifiers and buck–boost converters, to bridge the severe impedance mismatch between high-resistance outputs and standard low-impedance military batteries. Conversely, for self-powered sensing applications, the focus shifts to maximizing the signal-to-noise ratio and preserving waveform fidelity. These autonomous sensing nodes generally do not require backend energy storage circuits, but instead rely on high-input-impedance signal-conditioning modules to directly utilize real-time voltage or current waveforms as informational carriers, thereby enabling true zero-power stealth operation.

To systematically understand the diverse deployment of this technology, this review structures this section according to an escalating platform-scale and operational-domain framework, in which each specific use case is explicitly mapped to its corresponding functional circuit requirement. The applications are categorized into four overarching platform types. The first category focuses on the individual soldier combat platform, where biomechanical energy harvesting requires efficient power management to charge tactical radios, while tactical human–machine interfaces and physical layer information security operate as self-powered sensing nodes based on high-impedance signal processing. The second category addresses the unmanned combat platform, including environmental energy harvesting for auxiliary drone power and covert hydrodynamic perception for underwater operations, the latter functioning mainly as self-powered sensing. At a larger scale, the third category examines the strategic equipment platform, including auxiliary power supply for planetary exploration and passive structural monitoring of critical aerospace hardware, which depends primarily on sensing signal fidelity. Finally, the fourth category considers special tactical scenarios, including extreme operational conditions such as high-gravity weapon damage assessment, in which these devices function mainly as transient self-powered sensors. This hierarchical platform-based framework, combined with the explicit functional differentiation described above, clarifies both the technological evolution of TENG applications and the corresponding circuit-integration pathways required for practical military deployment.

### Individual Soldier Combat Platform

#### Physical Layer Information Security

In the highly digitized modern battlefield, the confidentiality of information and the reliability of identity authentication are directly related to the success or failure of military operations [138–142]. With the exponential growth of computing power, traditional encryption methods based on software algorithms are facing serious challenges from emerging technologies such as quantum computing. Building a new high ground of defense based on the physical properties of hardware has become an inevitable trend [143–145]. TENG provides a solution due to its extreme sensitivity to intrinsic material properties and mechanically triggered actions [146–148]. Unlike traditional biometrics are optical fingerprints or facial recognition, which rely predominantly on static geometric features vulnerable to high-resolution spoofing. Triboelectric material fingerprints represent complex dynamic electromechanical coupling, which synergistically integrates the inherent physical properties of dielectric materials with personalized behavioral biometric recognition by operators, creating a multidimensional dynamic encryption key that fundamentally resists static physical cloning.

**Fig. 4:**
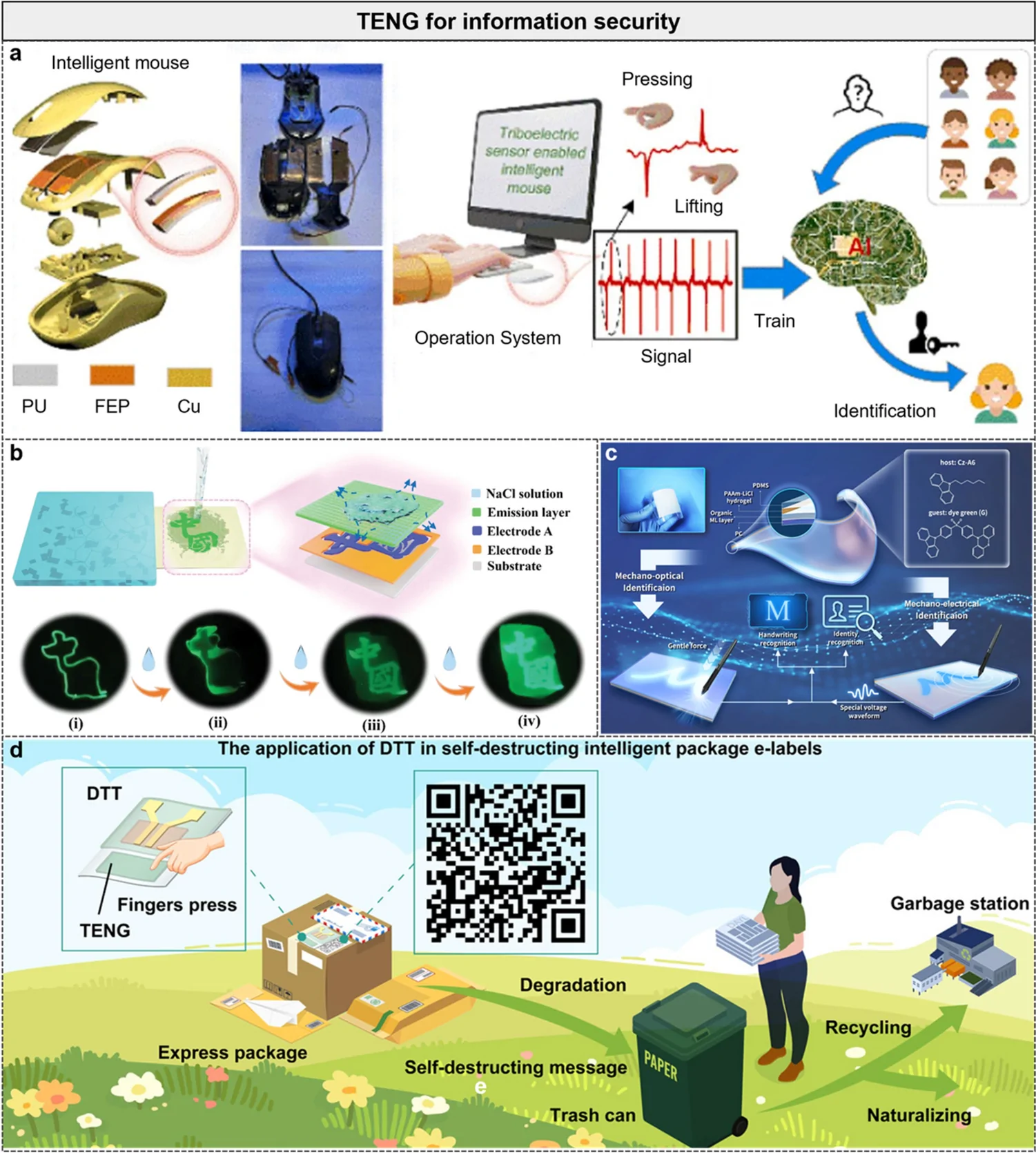
TENG for information security. **a** A security verification system that combines embedded intelligent mice, triboelectric sensors, and machine learning methods. Reproduced with permission from Ref. [149]. Copyright 2024, Elsevier. **b** Demonstration of device structure based on patterned coplanar electrodes and W-ELD encryption information. Reproduced with permission from Ref. [153]. Copyright 2023, Wiley–VCH. **c** Visual interactive tactile display (ITD) for dual-mode identification. Reproduced with permission from Ref. [154]. Copyright 2024, Wiley–VCH. **d** The information on the smart electronic tag will be self-destructed within its working cycle. Reproduced with permission from Ref. [155]. Copyright 2024, American Chemical Society

This distinctive mechanism makes TENGs particularly attractive for secure identity authentication on tactical terminals. The secure entry point of the combat command terminal is the first line of defense for information protection. Traditional static passwords or fingerprint recognition has the vulnerability of one-time authentication, valid throughout, and the consequences would be disastrous if the device is stolen by the enemy while unlocked. To address this, Zhang and his team proposed a dynamic defense strategy based on behavioral biometrics (Fig. 4a) [149]. This research embeds a TENG array into the surface of a mouse and combines it with machine learning algorithms to deeply analyze the subtle muscle tremors and force application habits of the operator during clicking and gripping. This behavioral fingerprint is difficult to imitate or physically replicate, and its core value lies in achieving unobtrusive continuous authentication with an accuracy rate of up to 98.4%: that is, without interrupting the commander’s operation, the system can monitor the operator’s identity in real time, and once abnormal behavioral characteristics are detected (such as operation by enemy personnel), it can block system access in milliseconds. This marks a leap in military terminal authentication from static passwords to dynamic behavior. However, the practical implementation of such triboelectric authentication systems in harsh military environments also raises a critical engineering challenge, namely the long-term stability of triboelectric signatures under humidity fluctuations, extreme temperatures, and continuous mechanical wear. To ensure operational reliability, these systems must combine robust material-engineering strategies, such as superhydrophobic surface modification and hermetic packaging, with adaptive backend algorithms that continuously update the authentication baseline, thereby compensating for gradual material degradation and signal drift during extended field deployment [150–152].

After identity authentication, how to conduct secure information interaction and display in silent or power-scarce field environments is another major pain point in tactical communication. Research in this field is evolving in two directions: structural and functional integration and dual-modal sensing. In the field of wearable interaction, Zhang et al. innovatively proposed an “All-in-One” integrated electrode strategy to address the problem of bulky individual equipment (Fig. 4b) [153]. By utilizing highly conductive and highly stretchable MXene/TPU nanofibers, they seamlessly integrated a TENG sensing layer with an electroluminescent (EL) display layer, constructing a flexible microsystem that can send Morse code via photoelectric signals with just a finger press. This design cleverly utilizes the mechanical–electrical–optical conversion mechanism, solving the problem of wearing comfort and providing a battery-free, completely silent tactical gesture communication method for special operations. Similarly, but with a different mechanism, Hou et al. developed a dual-modal tactile display using organic mechanoluminescent (OML) materials that do not require electrical excitation (Fig. 4c) [154]. Compared to EL devices, OML materials directly respond to mechanical pressure by emitting light. Combined with the electrical signals of handwriting recorded by TENG, it achieves dual authentication of what is written, what is seen, and what is written is stored. This technology effectively solves the problem that traditional pressure-sensitive screens cannot provide visual feedback after a power failure and is particularly suitable for signing and recording temporary combat orders on the battlefield.

When sensors or storage media face the extreme risk of being captured by the enemy, simple data deletion is often insufficient to completely eliminate the risk of leakage; physical destruction of the hardware itself becomes the last line of defense. Zhou’s research team deeply combined triboelectronics (an advanced technology utilizing the electrostatic potential generated by contact electrification to directly modulate charge transport in semiconductors) with biodegradable materials science to design a transient triboelectric transistor based on shellac and magnesium (Fig. 4d) [155]. This device exhibits excellent logic switching performance during its normal service life, but its physical structure and electronic function rapidly collapse and disappear upon receiving a specific environmental trigger. This breakthrough not only embodies the concept of green electronics but also has extremely high strategic significance in the military: it enables reconnaissance sensors or agent communication equipment deployed behind enemy lines to have the ability to disappear upon completion of the mission, completely cutting off the risk of intelligence leakage at the physical carrier level and building a closed loop of battlefield information security.

Overall, the application of TENGs in battlefield information security spans a coherent progression from identity authentication, to silent interaction and display, and finally to transient hardware destruction. This field exploits the unique nonlinear signals generated by micro- and nanoscale interfacial structures during contact electrification as a reliable source of physical entropy. Compared with software-based encryption, which may be challenged by algorithmic attacks, this hardware-rooted physical layer approach offers the natural advantages of non-replicability, concealment, and resistance to static cloning, making it highly attractive for zero-power and covert battlefield information protection. However, in realistic combat environments, adversaries may still attempt replay or spoofing attacks by recording and reproducing mechanical signatures, while machine-learning-assisted authentication systems can also be exposed to software-level vulnerabilities, such as model inversion or training-data leakage during tactical data transmission. Therefore, triboelectric physical entropy should not be regarded as a standalone security solution. Instead, the nonlinear physical signals generated during contact electrification should be further transformed into dynamic cryptographic keys and integrated with established system-level encryption protocols. Only by combining unique hardware-level physical traits with rigorous cryptographic standards can a genuinely robust, multilayered protection architecture be established for battlefield information security.

#### Wearable TENG-Enabled Devices for Energy Harvesting, Sensing, and Protection

With the rapid evolution of future individual soldier combat systems toward digitalization and all-domain awareness, individual soldier equipment is no longer merely a shield of physical protection, but has evolved into a complex microsystem integrating communication, navigation, and biochemical monitoring [156, 157]. However, the surge in electronic load has exacerbated the contradiction between energy supply and equipment weight [158–161]. While traditional TENGs have shown great potential in energy harvesting, conventional civilian materials often struggle to withstand the severe mechanical shocks, chemical corrosion, extreme temperature changes, and strong electromagnetic interference of the battlefield environment [162–165]. Therefore, developing structure function-integrated triboelectric materials specifically for military scenarios has become a current research frontier [166, 167]. Furthermore, various 3D printing technologies, such as fused deposition modeling and stereolithography, enable the precise fabrication of complex internal structures, significantly facilitating the mass production of these flexible military wearable devices [168]. Together with biomimetic design, these advanced manufacturing capabilities provide an important basis for understanding the rapid development of technologies in this field.

**Fig. 5:**
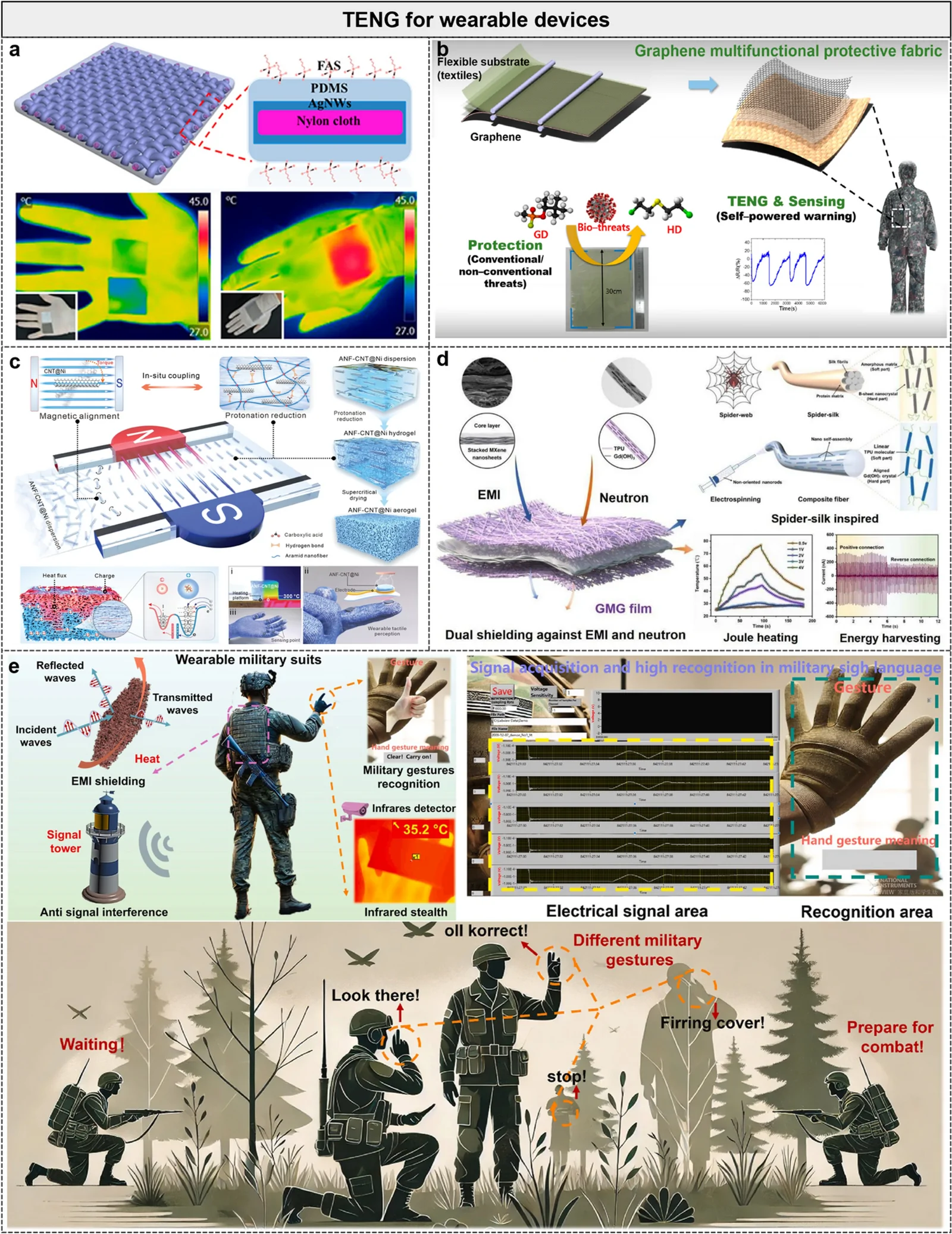
TENG for wearable devices. **a** Textile personal energy management device improved by fluoroalkylsilane. Reproduced with permission from Ref. [169]. Copyright 2016, American Chemical Society. **b** Schematics of graphene multifunctional protective fabrics. Reproduced with permission from Ref. [170]. Copyright 2021, MDPI. **c** Schematic illustration of preparation and functional design of anisotropic structural triboelectric aerogels. Reproduced with permission from Ref. [171]. Copyright 2023, Wiley–VCH. **d** A multifunctional wearable spider silk-inspired fabric suitable for personal protection in extreme environments. Reproduced with permission from Ref. [172]. Copyright 2024, Elsevier. **e** Composite foams as wearable military suits with multifunctional applications. Reproduced with permission from Ref. [173]. Copyright 2025, Elsevier

The primary hurdle in introducing TENGs into individual soldier equipment is solving their robustness problem in complex battlefield environments. Conventional fabric TENGs are highly susceptible to charge dissipation due to sweat and high humidity, and lack water resistance, making them unsuitable for field service requirements. To address this pain point, Guo and his team pioneered the technical route of surface chemical modification [169]. They constructed a self-assembled monolayer on the surface of fabric fibers using fluoroalkylsilanes (FAS), as shown in Fig. 5a. Leveraging the high electronegativity and low surface energy of fluorine groups, they achieved a dual breakthrough in improving electron affinity and superhydrophobic self-cleaning functionality. This work not only solved the energy conversion efficiency problem but, more importantly, verified the core value of a chemical protective layer in maintaining the stable operation of TENGs in humid, sweaty, and muddy environments, laying the engineering foundation for all-weather wearable energy systems. Building on this, facing the more lethal biological and chemical threat, simple physical protection is insufficient; endowing equipment with real-time sensing capabilities against nerve agents and other chemical warfare agents has become a new strategic requirement. Jin et al. further expanded the functional boundaries of smart fabrics, achieving the non-destructive transfer of high-quality chemical vapor deposition graphene to rough fabrics using an ethylene − vinyl acetate hot-melt lamination process (Fig. 5b) [170]. This design not only constructed flexible electrodes resistant to thousands of bends but also integrated chemical sensors with high sensitivity to nerve agents. The significance lies in demonstrating that graphene materials can upgrade traditional passive chemical protective suits into active defense systems with electronic nose functionality, significantly reducing the weight carried by individual soldiers while achieving early warning of stealthy chemical threats. Besides chemical threats, the shockwaves from explosions and the high-temperature heat flow generated at fire scenes are another major cause of death for individual soldiers. Traditional polymer-based TENGs often experience rapid charge dissipation at high temperatures due to thermionic emission, thus failing at critical moments when vital signs monitoring is most needed. To address this physical bottleneck, Chi and his team innovatively introduced a magnetic field-assisted modulation strategy to prepare anisotropic aramid triboelectric gels (Fig. 5c) [171]. Through in situ coupled magnetic orientation technology, aramid nanofibers were arranged in a highly ordered manner along specific directions, constructing a microscopic barrier capable of blocking the thermal escape of electrons. This groundbreaking technology enables devices to maintain an astonishing charge density (75 μC m⁻^2^) even at extreme temperatures of 300 °C.

As modern warfare shifts toward information-based and highly confrontational approaches, individual soldier equipment must not only protect against physical attacks but also cope with multi-dimensional threats such as neutron radiation, electromagnetic interference, and infrared reconnaissance. To address this all-encompassing challenge, materials design must leap toward multi-functional synergy. Inspired by the microstructure of spider silk, Zhu and his team proposed a biomimetic design concept based on the synergy of soft and hard segments (Fig. 5d) [172]. By introducing Gd(OH)_3_ nanorods as crystalline hard segments to reinforce the TPU matrix and combining them with an MXene core layer, they constructed a sandwich smart fabric integrating high strength and toughness, neutron/electromagnetic dual shielding, and optical/electrothermal management. Furthermore, addressing the urgent needs of special operations for multi-spectral stealth and silent communication, Xu et al. focused on dual stealth in both the electromagnetic and infrared bands (Fig. 5e) [173]. They fabricated a multifunctional composite foam (PU-CA) that combines lightweight (0.07 g cm^−3^) and high conductivity by constructing a dual conductive network of carbon nanotubes (CNTs) and silver nanowires (AgNWs). Its unique porous structure and conductive network synergistically achieved an excellent electromagnetic shielding effectiveness of 50.12 dB in the X-band, while significantly suppressing thermal radiation signals by reducing surface infrared emissivity, thus achieving dual electromagnetic and infrared camouflage. At the tactical application level, the team used this composite foam to construct a highly sensitive (0.39 V kPa^−1^) single-electrode TENG sensor and integrated it into a smart tactical glove. This system can not only capture the triboelectric signals generated by finger bending in real time, but also accurately identify military hand gestures such as “stop” and “cover” using algorithms, enabling silent battlefield command transmission under passive conditions. This work ingeniously integrates passive multidimensional stealth protection with active self-powered tactical awareness, providing an important materials science paradigm for the multi-functional integration of future digital individual combat systems.

In the field of individual equipment, a common breakthrough enabled by triboelectric technology lies in the exceptional environmental adaptability provided by microscale and nanoscale surface engineering. To provide a clearer engineering roadmap for future tactical equipment development, it is essential to quantitatively evaluate the performance trade-offs among different material modification strategies. As summarized in Table 2, the construction of fluorinated nanoarrays or conductive composite networks on fiber surfaces not only addresses the critical weaknesses of traditional chemical batteries, such as low-temperature failure and impact-induced explosion, but also enables the seamless integration of energy modules with protective equipment. A comparative quantitative analysis further shows that, although surface fluorination effectively mitigates charge dissipation under high humidity, the thermal tolerance of conventional polymers often remains a major bottleneck. To overcome this limitation, the introduction of magnetically assisted aramid aerogels extends the operational temperature limit to 300 °C while maintaining robust charge density. Furthermore, for applications requiring extreme mechanical durability and multispectral stealth, composite networks based on chemical-vapor-deposited graphene or MXene exhibit superior performance by preserving structural and electrical integrity over thousands of continuous bending cycles. This combination of passive protection and active sensing, achieved through targeted material modification, is difficult to realize with traditional discrete sensor devices. Overall, this quantitative comparison indicates that future materials design must balance thermal stability, mechanical endurance, and environmental resilience more precisely in order to improve the survivability of individual soldiers in complex battlefield environments.

**Table 2:** Key performance metrics comparison of wearable TENG material systems with different modification strategies

Modification strategy	Method	Humidity test	Durability	Temperature tolerance	Refs
Fluoroalkylsilane (FAS) grafting	Dip-coating (PDMS/AgNWs/nylon)	Contact angle 131°	12,000 cycles	37–80 °C	[169]
Hot-melt lamination transfer	Co-lamination (graphene transfer onto fabric/EVA)	–	1000 bending cycles	–	[170]
Magnetic field-induced directional assembly	Magnetic alignment and protonation reduction (anisotropic ANF/CNT@Ni aerogel)	–	2200 cycles	50–300 °C	[171]
Structural bionic design	Electrospinning and vacuum filtration (Gd(OH)_3_/TPU-MXene)	–	Tensile strength 22.94 MPa	Joule heating up to 78.5 °C	[172]
Double conductive network	Electroplating and vacuum-assisted dip-coating (CNT/AgNW)	20–60% RH	10,000 cycles at 2 Hz	0–40 °C	[173]

#### Tactical Human–Machine Interface and Vital Monitoring

In the macrocontext of digital warfare and human–machine collaborative combat, building efficient and intuitive human–machine interaction (HMI) systems has become a key link between individual intelligence and unmanned equipment [174]. However, traditional handheld controllers not only significantly increase the burden on individual soldiers, but also severely limit their tactical mobility in high-pressure environments such as encounter battles due to occupying soldiers’ hands [175]. The self-powered flexible sensing technology based on frictional nanogenerators provides a breakthrough solution for the next generation of tactical HMI, thanks to its unique zero-power consumption characteristics and excellent structural adaptability [176–181].

**Fig. 6:**
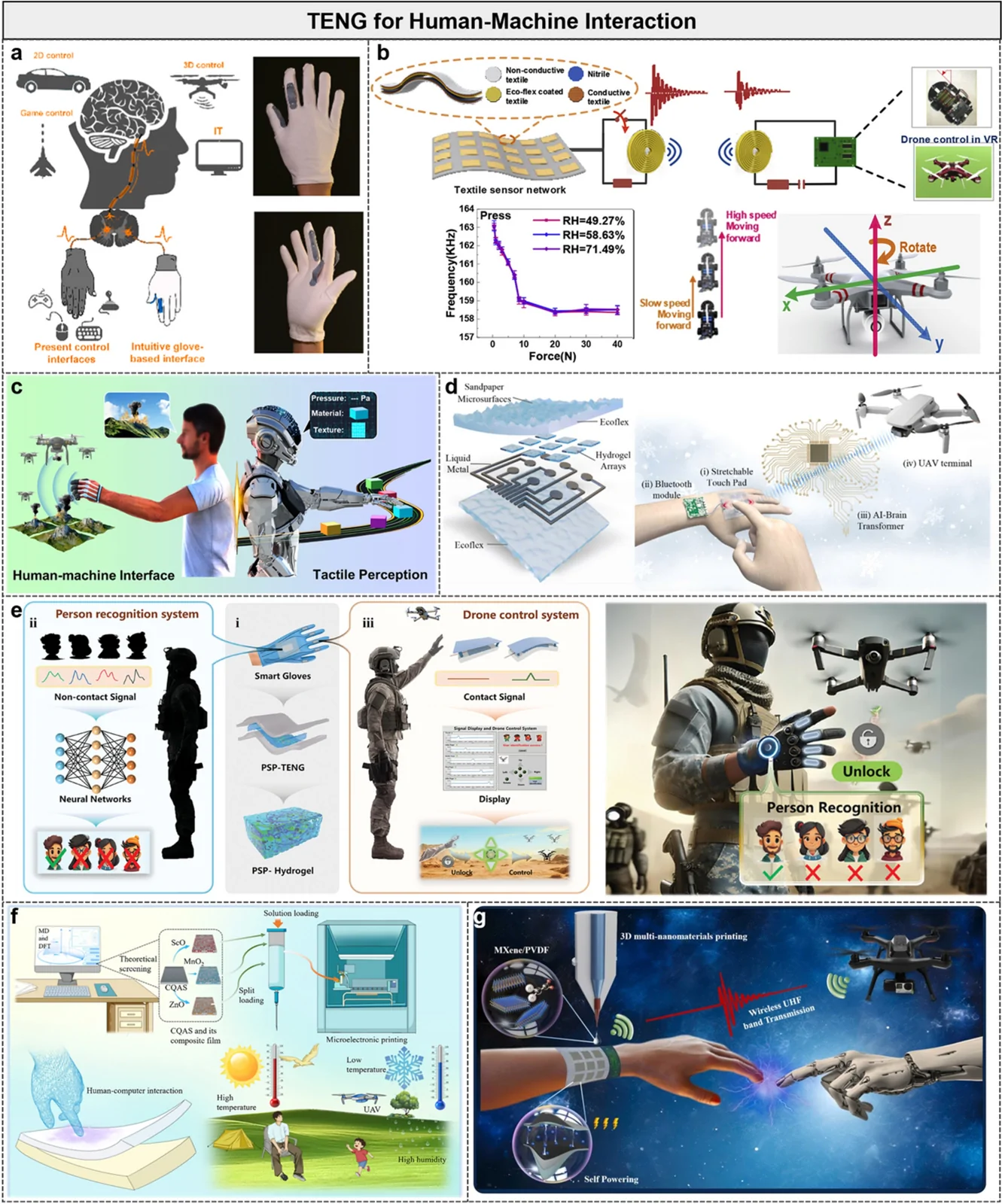
TENG for human–computer interaction. **a** Minimalist design of a glove-based HMI based on the TENGs in two configurations. Reproduced with permission from Ref. [182]. Copyright 2019, Elsevier. **b** Wireless TENG system for car control and drone control in VR based on an RLC circuit. Reproduced with permission from Ref. [183]. Copyright 2020, Elsevier. **c** Application scenarios of BHES as a human–machine interaction interface. Reproduced with permission from Ref. [184]. Copyright 2023, American Chemical Society. **d** Stretchable triboelectric touch pad structure and an intelligent human–machine interaction system. Reproduced with permission from Ref. [185]. Copyright 2024, Elsevier. **e** PSP-TENG is integrated into smart gloves and its application scenarios in face-to-face recognition and drone control. Reproduced with permission from Ref. [186]. Copyright 2024, Wiley–VCH. **f** Realize HMI through CQAS/ZnO thin films and promote the development of drones. Reproduced with permission from Ref. [187]. Copyright 2025, Wiley–VCH. **g** Design and conceptual illustration of MXTENG-RFS. Reproduced with permission from Ref. [188]. Copyright 2025, Elsevier

Returning control from cumbersome terminals to the soldier’s limb movements is the primary evolutionary direction of tactical HMIs. He and his team pioneered this path, designing a fully fabric triboelectric sensing smart glove (Fig. 6a) [182]. This research seamlessly stitches flexible TENGs to the fingertips and palm, utilizing self-powered pulses generated by finger bending to directly map gestures into control commands for drones or robotic arms. This minimalist hardware solution, allowing for wearable control, completely frees soldiers’ hands, making it possible to issue commands while on the move by waving their hands. Besides single-point control, building a distributed battlefield awareness network is equally crucial. Addressing the difficulty of maintaining traditional sensor nodes, Wen et al. proposed a battery-free wireless network solution based on Direct Sensing Transmission (Fig. 6b) [183]. It utilizes high-voltage pulses generated instantaneously by a TENG to directly drive an infrared-emitting unit, achieving zero-power communication that activates upon pressing. This minimalist architecture provides an ideal paradigm for building disposable tactical sensor networks, making it highly suitable for rapidly deploying maintenance-free triggers or silent communication nodes in field environments.

Real battlefield environments are far harsher than laboratory environments. High humidity, severe deformation, and mechanical damage are the core bottlenecks restricting the reliability of HMI equipment. Researchers have engaged in in-depth research and development at the material and algorithm levels to address these challenges. To give the robot more refined tactile perception, Tao and his team designed a biomimetic multifunctional electronic skin (BHES) (Fig. 6c) [184]. By mimicking the layered structure of dermis/epidermis, integrating triboelectric and piezoresistive mechanisms, and combining deep learning, they achieved a full-dimensional perception of materials, textures, and pressure. In dealing with extreme deformation, Liu et al. combined liquid metal with sandpaper molding technology to develop a highly stretchable triboelectric touchpad (Fig. 6d) [185]. To address the challenge of nonlinear signal drift under high strain, this research introduced a transformer deep learning model, which automatically corrects signal distortion caused by deformation through an attention mechanism. This strategy ensures that soldiers can accurately input handwritten commands even during strenuous tactical maneuvers such as crawling and climbing. In terms of addressing mechanical damage and safety, Wang et al. developed a smart glove based on a self-healing hydrogel (Fig. 6e) [186]. This system not only enables rapid self-repair after physical damage, but also integrates a biometric recognition algorithm based on aerial handwriting trajectories. This ingenious design transforms the operator's hand gestures into a dynamic password, creating a dual line of defense for individual unmanned systems, combining physical resilience and access control. Regarding high-humidity environments, Zheng and his team proposed a modification strategy based on the electrostatic attraction between biomass and metal oxides at the molecular level (Fig. 6f) [187]. By introducing deep-level defects in ZnO nanoparticles to trap electrons, charge dissipation in humid environments was significantly suppressed. This allowed the sensor to output a voltage as high as 1260 V even in a simulated tropical rainforest environment, effectively solving the problem of performance degradation of self-powered controllers in hot and humid combat zones.

With the escalation of electronic warfare threats, reducing the electromagnetic signature and size of wearable devices has become a new strategic priority. Shingirirai et al. have pushed HMI technology to the ultimate stealth form. They designed a wireless electronic skin with completely active-electronic-free sensor components (Fig. 6g) [188]. By directly modulating passive radio-frequency circuits using TENG, they completely eliminated the battery, transistors, and chips at the sensor end. This architecture not only achieves extreme thinness and flexibility, but also, because it lacks active electronic components, is extremely difficult for enemy electronic reconnaissance equipment to locate. It provides special forces with a stealth tattoo-like control interface, achieving dual concealment of human–computer interaction at both the physical and electromagnetic levels.

The innovation in the field of human–computer interaction is mainly due to the ultra-high-sensitivity response of TENG micro-/nanostructures to small mechanical deformations. Compared to traditional potentiometers or inertial controllers that require an external power supply and are bulky, TENG-based flexible micro-/nanosensors can closely adhere to the skin surface, converting subtle muscle tremors or joint bends into precise control commands. Its irreplaceability lies in the realization of passive and flexible control circuits, which greatly reduce the burden on individual soldiers while providing silent tactile feedback and concealed operation capabilities that traditional mechanical switches cannot achieve.

### Unmanned Combat Platform

#### Underwater Covert Operations and Biomimetic Perception

Underwater covert operations require absolute stealth, making traditional active sonar systems increasingly vulnerable to enemy detection. To overcome this limitation, unmanned underwater vehicles urgently need passive perception networks that do not emit acoustic signals. Triboelectric technology offers an attractive solution by directly converting environmental mechanical energy into electrical signals, thereby creating a completely silent and self-powered sensory system. On this basis, a relatively complete underwater perception architecture can be established, extending from far-field vibration detection and near-field hydrodynamic monitoring to high-frequency acoustic sensing and the internal proprioception of biomimetic propulsion.

**Fig. 7:**
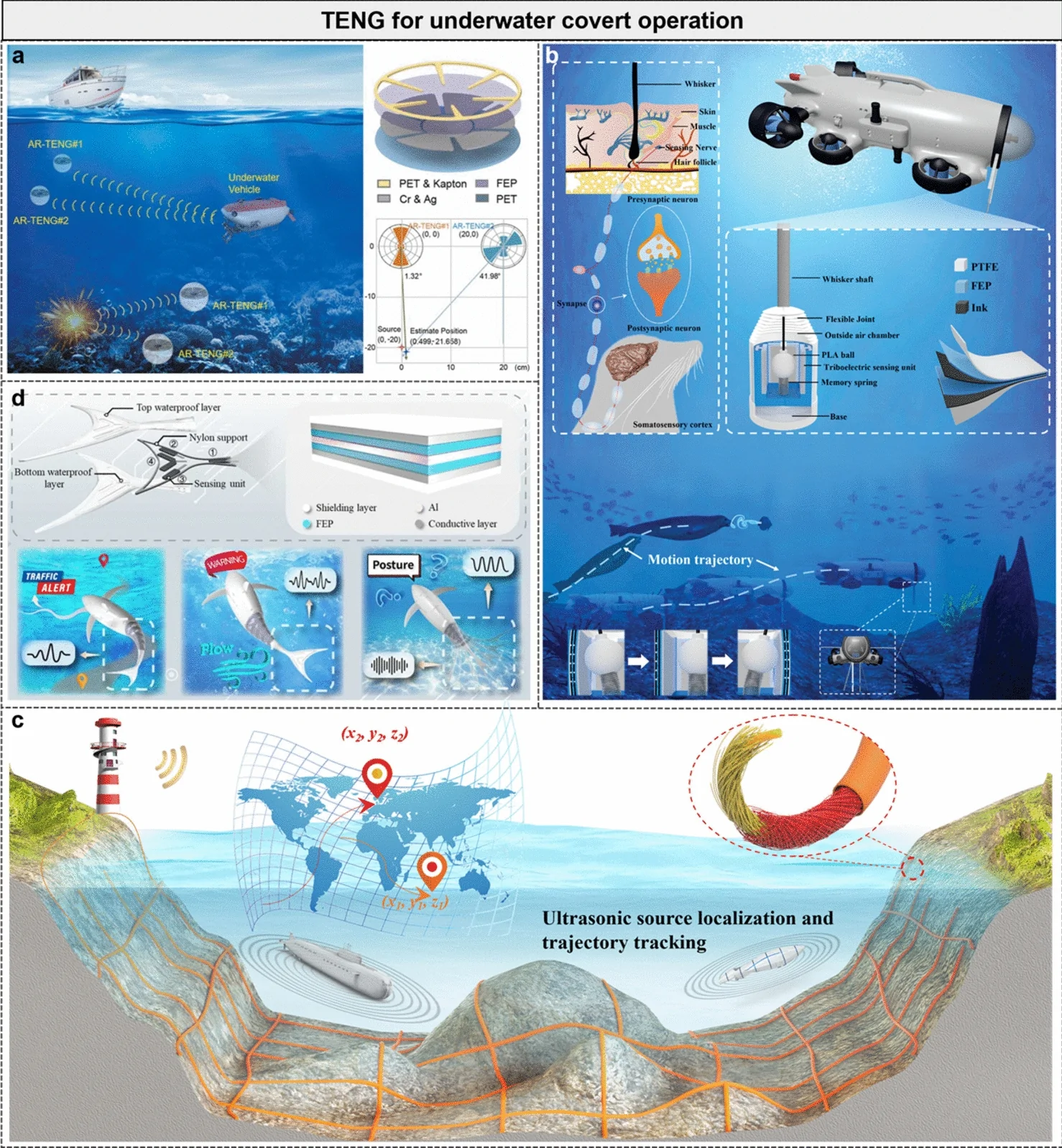
TENG for underwater covert operation. **a** A self-powered angle-resolved triboelectric nanogenerator. Reproduced with permission from Ref. [189]. Copyright 2023, Elsevier. **b** A bionic underwater triboelectric whisker sensor array. Reproduced with permission from Ref. [190]. Copyright 2024, Wiley–VCH. **c** Schematic representation of a self-powered ultrasonic source localization and trajectory tracking system, incorporating multiple MTAS units deployed in the ocean. Reproduced with permission from Ref. [121]. Copyright 2025, American Chemical Society. **d** Working principles, structures, and application of the biomimetic tail fin sensor. Reproduced with permission from Ref. [191]. Copyright 2026, Elsevier

As the first step in this architecture, far-field vibration detection provides crucial early-warning capabilities. Inspired by the lateral line systems of aquatic animals, Guo developed an angle-resolved triboelectric nanogenerator to precisely locate underwater vibration sources (Fig. 7a) [189]. Based on a solid–liquid contact electrification mechanism, this sensor continuously monitors the frequency and amplitude of low-frequency water disturbances. By utilizing a multi-unit sensor array, it can accurately calculate the spatial coordinates of external targets. This passive detection capability is highly valuable for the covert tracking of enemy submarines. Compared with easily traceable active sonar, this self-powered strategy ensures continuous threat awareness while strictly maintaining the acoustic stealth of the underwater platform.

Building upon this far-field perception of external threats, the dynamic motion control of unmanned underwater vehicles requires precise real-time near-field hydrodynamic monitoring. To address this complex fluid–solid interaction, Liu et al. engineered a deep-learning-assisted triboelectric whisker sensor array intended to capture subtle changes in localized water-flow velocity and direction (Fig. 7b) [190]. Constructed from highly durable polytetrafluoroethylene and copper wires, this bionic whisker system dynamically interacts with the turbulent underwater environment. By coupling the multidimensional triboelectric signals with a sophisticated one-dimensional convolutional neural network, the integrated system achieved a remarkable state-recognition accuracy exceeding 98% for various complex vehicle maneuvers, including forward, backward, and rapid steering motions. This seamless integration of self-powered hardware and artificial intelligence effectively addresses the inherent limitations of traditional optical sensors in turbid waters, offering a highly robust navigational alternative when conventional satellite signals are entirely unavailable in deep-sea environments.

While low-frequency hydrodynamic perception secures local physical navigation, high-frequency acoustic sensing remains indispensable for broader secure communication and coordinated swarm positioning. To address the severe attenuation of traditional electromagnetic waves in aquatic environments, Dong et al. developed an advanced microfiber-based triboelectric acoustic sensor employing electrospun polyvinylidene fluoride and highly conductive MXene composites (Fig. 7c) [121]. This specialized device overcomes the traditional low-frequency limitation of triboelectric materials by exhibiting exceptional structural sensitivity and a broad operational bandwidth, making it highly capable of accurately capturing high-frequency ultrasonic signals. By utilizing precise time-of-flight measurement principles, the acoustic sensor array enables highly accurate underwater target tracking and spatial positioning without relying on bulky external power supplies. Strategically, this advanced ultrasonic capability is instrumental in coordinating covert swarms of autonomous underwater vehicles, ensuring secure tactical communication networks while maintaining strict energy independence over extended mission durations.

At a deeper level of underwater stealth, biomimetic robotic fish represent a further evolution in platform design, but their complex undulating propulsion imposes stringent demands on internal proprioception. To meet this requirement, Liu and associates fabricated a multi-degree-of-freedom semi-flexible embedded bionic tail-fin sensor utilizing liquid metal electrodes and highly stretchable elastomer matrices (Fig. 7d) [191]. This sophisticated solid-state sensor accurately monitors the localized bending and torsional deformations of the biomimetic tail fin during rapid propulsion. When directly integrated with machine-learning algorithms, it precisely recognizes the instantaneous swimming state and tail-beat frequency, thereby providing crucial real-time feedback for closed-loop motion control. Importantly, the embedded configuration avoids disrupting the original hydrodynamic profile of the robotic fish. As a result, the platform can more closely mimic biological swimming patterns, thereby reducing hydrodynamic wake and suppressing the acoustic signature that could otherwise be exposed to enemy sonar systems.

Overall, the deployment of triboelectric technology in underwater environments is forming a progressively integrated covert perception framework. This framework spans from passive sensing of external vibration signals to local hydrodynamic navigation, to high-frequency acoustic communication and positioning, and finally to embedded proprioception in biomimetic propulsion systems. Across these different levels, self-powered triboelectric devices reduce dependence on bulky battery modules and avoid the active signal emission associated with conventional sensing technologies. More importantly, this evolution from isolated sensor units to intelligent perception networks supported by data processing and machine learning points to a new paradigm for autonomous marine operations. It not only extends the endurance of unmanned underwater systems but also enhances their acoustic and electromagnetic stealth, thereby providing a promising technological route for the next generation of deep-sea tactical platforms.

#### Aerodynamic Harvesting and Self-Powered Flight Perception

In the modern information-driven battlefield and the architecture of the military Internet of Things, unmanned aerial vehicles (UAVs) have become a core node for performing reconnaissance and precision strike missions [192, 193]. However, their overall combat effectiveness has long been limited by size, weight, and power consumption bottlenecks, as well as battery life constraints [194, 195]. TENGs, with their lightweight materials, high-voltage output, and excellent structural adaptability, provide a revolutionary technological path to overcome these limitations [196–198]. They can not only convert mechanical energy during flight into electrical energy, enabling environmental energy harvesting during silent operation, but also endow UAVs with self-powered, bio-inspired neural sensing capabilities [199, 200].

**Fig. 8:**
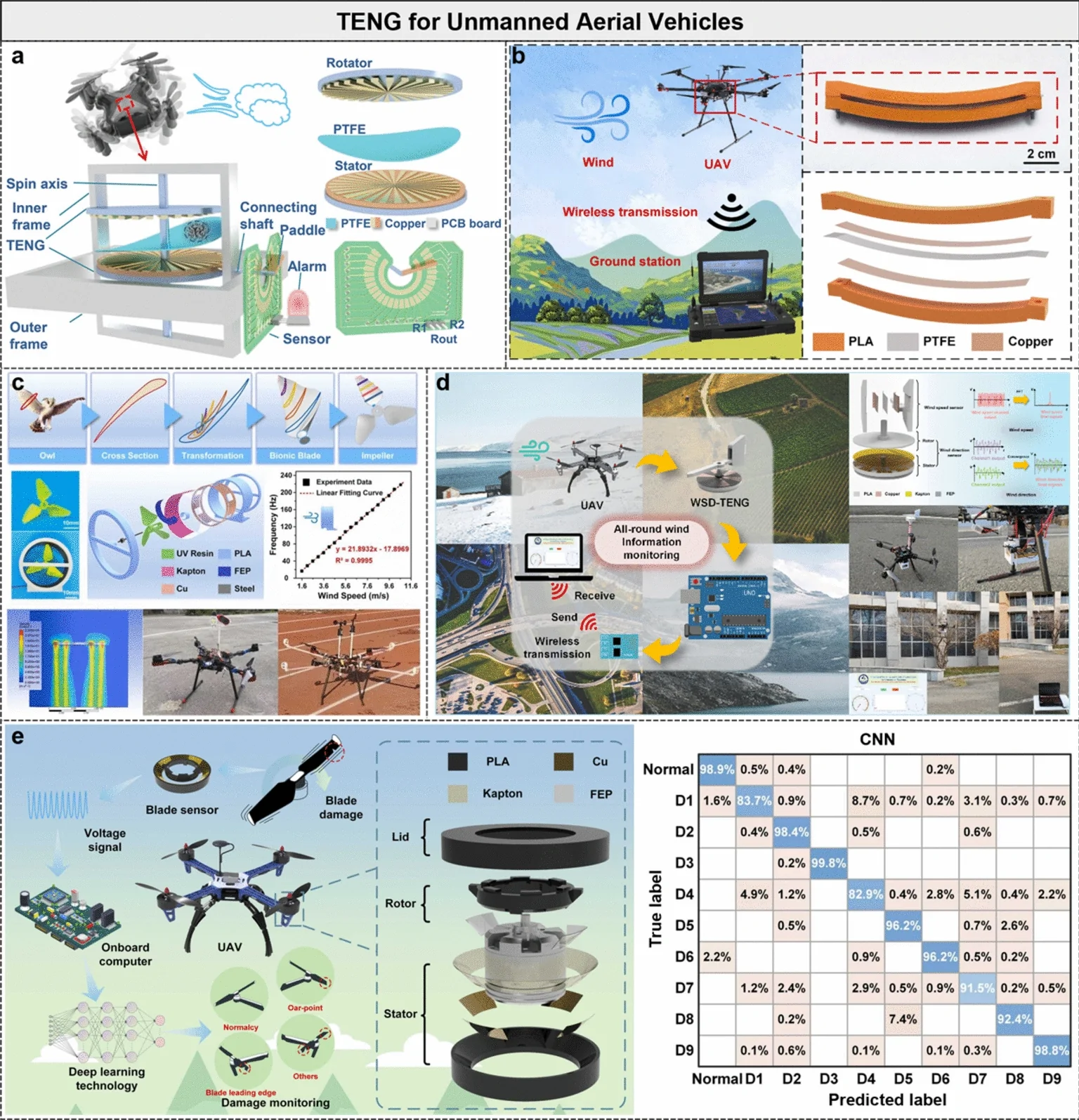
TENG for Unmanned Aerial Vehicles. **a** Self-powered gyroscope angle-sensing system. Reproduced with permission from Ref. [201]. Copyright 2021, Wiley–VCH. **b** Structure and application of AW-TENG. Reproduced with permission from Ref. [202]. Copyright 2022, Elsevier. **c** Structural design and application of BOW-TENG. Reproduced with permission from Ref. [203]. Copyright 2024, Elsevier. **d** Structural design and application of the WSD-TENG. Reproduced with permission from Ref. [204]. Copyright 2025, American Chemical Society. **e** UAV blade damage monitoring system based on a triboelectric sensor. Reproduced with permission from Ref. [205]. Copyright 2025, Elsevier

Real-time stabilization of flight attitude is the cornerstone of UAV mission execution. While traditional gyroscopes and accelerometers offer mature accuracy, their high power consumption and reliance on complex demodulation circuits become a vulnerability in extreme conditions, such as silent standby or main power failure in miniature UAVs. To address this issue, Xie et al. proposed a self-powered gyroscopic angle sensor (SGAS) based on the resistance matching effect (Fig. 8a) [201]. This research did not follow the traditional TENG “pulse counting” digital measurement logic, but cleverly utilized the physical characteristic of TENG’s internal resistance, linearly changing with contact area, directly mapping the mechanical rotation angle to an analog change in load voltage. This design eliminates complex signal demodulation circuits, achieving high-sensitivity monitoring of 67.3 mV/deg and fast response monitoring of less than 20 ms. Its strategic value lies in maintaining a basic perception of the aircraft's attitude even in the event of main power failure or silent standby, improving battlefield survivability, and the ability to resume flight.

In addition to body attitude, real-time perception of complex airflow environments is crucial for ensuring stable formation flight and performing meteorological reconnaissance missions in turbulent conditions. In this field, TENG sensors have undergone a technological evolution from single-mechanism verification to bionic enhancement and then to multidimensional integration. Early explorations focused on lightweight design. Yao et al. designed an arc-shaped flutter-driven wind speed sensor (AW-TENG), utilizing aeroelastic flutter induced by fluid flow over an arc-shaped surface to drive thin-film power generation (Fig. 8b) [202]. They successfully established a linear relationship between signal frequency and wind speed, solving the problem of excessive load on traditional anemometers. However, facing the low wind speed or calm atmospheric conditions common in battlefield reconnaissance, the high mechanical starting threshold became a new bottleneck. To address this, Liu et al. introduced a bionic design, inspired by the high-lift airfoil of owl wings (Fig. 8c) [203]. Through CFD simulation, they optimized the impeller curvature, significantly increasing the driving torque at low flow velocities. This bionic improvement not only greatly reduced the sensor's cut-in wind speed but also extended the lower limit of its linear measurement range, enabling high-altitude, long-endurance UAVs to obtain accurate meteorological data during stratospheric reconnaissance. To further meet the battlefield's need for comprehensive airflow vector information, Wang et al. broke through the limitations of single-parameter measurement and proposed a triboelectric omnidirectional adaptive sensor with a hierarchical structure (WSD-TENG) [204], as shown in Fig. 8d. This design cleverly uses a coaxial hierarchical architecture, physically integrating the wind cup speed measurement in the upper layer with the wind vane encoding direction measurement in the lower layer, eliminating signal crosstalk while significantly reducing the windward area. This integrated design is of great significance for shipborne or field-deployed UAVs, assisting flight control systems in performing millisecond-level attitude compensation under complex crosswind or gust conditions. With the intervention of artificial intelligence, TENG is evolving from simple signal acquisition to intelligent diagnosis in the field of fault diagnosis. Pan et al. demonstrated the unique advantages of TENG signals as an AI data source, using high signal-to-noise ratio vibration waveforms collected by TENG, combined with a convolutional neural network, to achieve accurate classification of minute damage on propeller blades (Fig. 8e) [205].

In unmanned aerial vehicles applications, the core advantage of triboelectric nanogenerators lies in their efficient capture of low-frequency aerodynamic energy and wideband response for self-powered sensing. Compared to traditional anemometers or external piezoelectric sensors that significantly increase aerodynamic drag, these devices can be directly integrated onto the wing surface using micro- and nanolithography technology to achieve real-time monitoring without changing the aerodynamic shape. While this integrated microdesign enhances the long-endurance capabilities of drone swarms, interfacing these high impedances pulsed alternating current signals with existing unmanned aerial vehicle avionics presents a critical practical challenge. Standard flight control systems typically require stable, low-impedance direct current inputs to process telemetry and navigational data. As will be systematically discussed later in the power management dilemmas of Sect. 4.1, bridging this fundamental electrical gap necessitates specific interface strategies. To prevent severe signal attenuation, specialized signal-conditioning circuits must be inserted between the sensor arrays and the main avionics bus. Alternatively, researchers are developing innovative electromechanical designs that naturally match the circuit impedance to directly output measurable analog voltages. These practical interface solutions effectively eliminate the need for overly complex backend circuits, thereby facilitating seamless electrical integration with existing flight computers and successfully bridging the gap between advanced material perception and conventional aerospace avionics.

### Strategic Equipment Platform

#### TENG-Enabled Devices for Aerospace Equipment Monitoring

Large-scale strategic aerospace equipment is the commanding height of modern national defense systems [206]. The reliability of their core components directly affects the success or failure of missions and the safety of high-value assets [207, 208]. However, facing the high-temperature oil mist shielding within aero-engine casings, the narrow non-guided gaps in spacecraft flywheel assemblies, and the extreme microgravity environment of on-orbit operations in space, traditional active sensors are limited by physical bottlenecks such as power cables cannot be routed in, and wireless signals cannot be transmitted, making it difficult to achieve real-time access to the deep lesions of equipment [209–211]. TENG, with its unique advantage of integrated structure and function, is triggering a paradigm revolution from passive periodic maintenance to proactive condition-based maintenance [212–214].

**Fig. 9:**
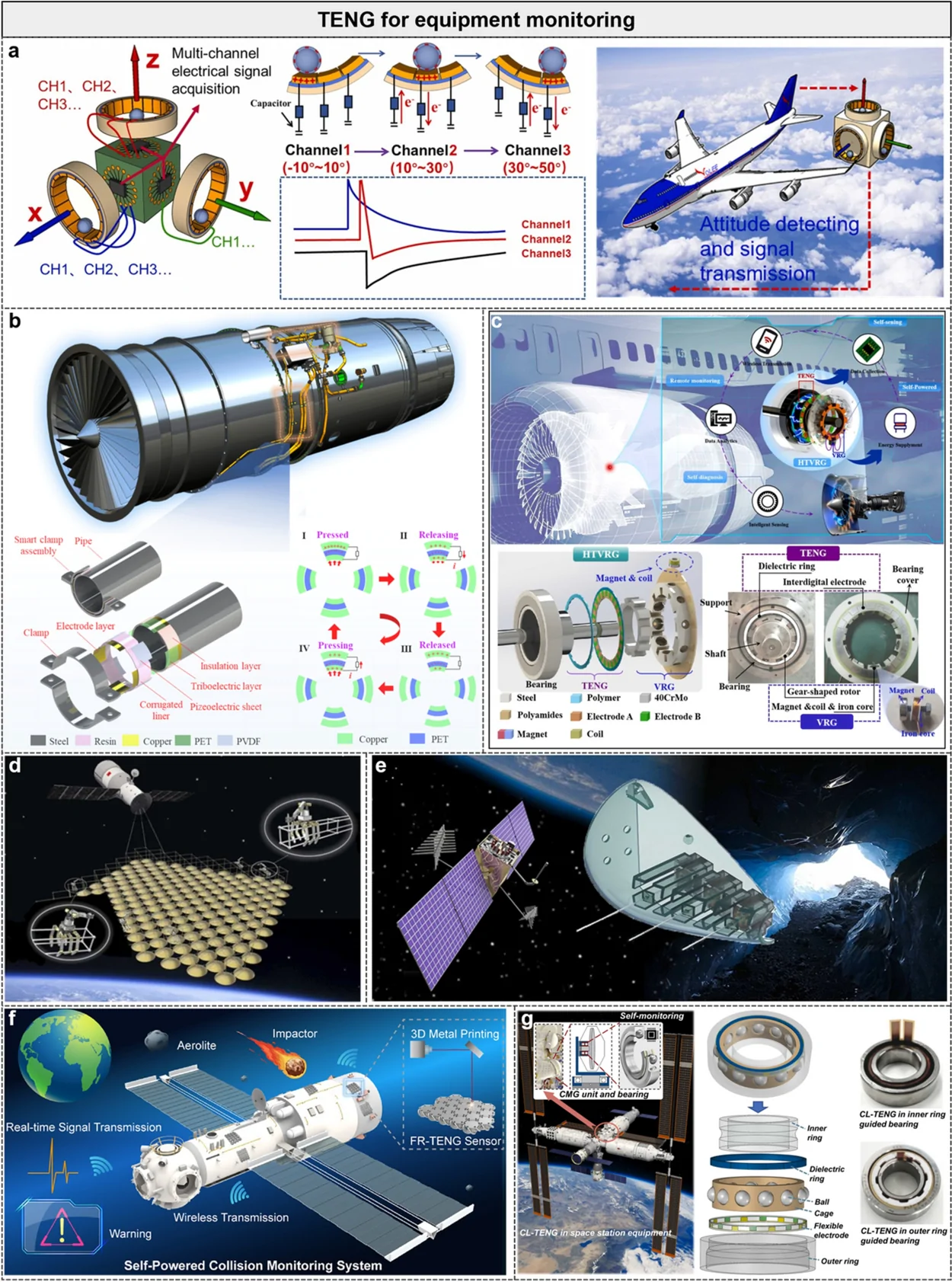
TENG for equipment monitoring. **a** Design diagram and working schematic of a multi-channel self-powered sensor. Reproduced with permission from Ref. [215]. Copyright 2023, Elsevier. **b** Structural design and operating mechanisms of TPPC. Reproduced with permission from Ref. [216]. Copyright 2025, Elsevier. **c** Application prospects and structure of the HTVRG. Reproduced with permission from Ref. [217]. Copyright 2025, Elsevier. **d** Schematics of scalable self-attaching/assembling robotic cluster system. Reproduced with permission from Ref. [218]. Copyright 2022, Elsevier. **e** Application of BMWS for satellite surface feature acquisition in dark environments. Reproduced with permission from Ref. [219]. Copyright 2023, Elsevier. **f** Design and concept of the robust but flexible FR-TENG array is inspired by hexagon and pangolin scales. Reproduced with permission from Ref. [220]. Copyright 2023, American Chemical Society. **g** Application background and structure of the bearing-based CL-TENG. Reproduced with permission from Ref. [221]. Copyright 2024, Elsevier

The attitude perception of aircraft is the cornerstone of navigation and control. Wang et al. proposed a multi-channel self-powered attitude sensor based on the coupling of TENGs and inertia principles to address the pain points of traditional inertial measurement units failing under extreme working conditions, such as power outages or electronic system failures (Fig. 9a) [215]. This study adopted a clever three-dimensional orthogonal structure design, with circular tracks set on the three faces of the cube. Steel balls wrapped in FEP were used as inertial mass blocks inside. When the aircraft tilts or rotates, the steel balls roll along the track under the action of gravity and inertia, sweeping through 18 independent electrode channels distributed on the track and generating discrete frictional electrical signals. By analyzing the triggered channel number and signal timing, the system can reconstruct the pitch, roll, and yaw attitude of the object in three-dimensional space in real time. This pure physical structure sensor can work without any power supply, and the multi-channel design naturally has anti-interference ability, which can accurately map the angle changes of analog signals in a digital way. This characteristic of maintaining basic navigation functions under passive conditions provides a highly promising solution for the last line of defense of aircraft after strong electromagnetic countermeasures or energy depletion.

From the flight attitude to the power system, the hydraulic and fuel pipelines densely distributed outside the aircraft engine are like the “blood vessels” of the aircraft. The loosening of clamps and fatigue cracks under long-term severe vibration are hidden dangers that can trigger catastrophic failures. Zhu et al. proposed a design concept of “structure as sensor” to address the challenges of large volume and wiring required for traditional external sensors. They did not introduce additional sensing probes, but integrated TENG and piezoelectric elements into standard pipeline clamp pads, constructing a triboelectric piezoelectric composite intelligent pipeline clamp (TPPC) (Fig. 9b) [216]. This design cleverly utilizes the sensitivity of hybrid generators to small modal changes in vibration: TENG is sensitive to low-frequency, large-amplitude shaking, while piezoelectric modules are adept at capturing high-frequency, small-amplitude vibrations. Combined with the 1D ResNet architecture, the system achieved a classification accuracy of up to 99.76% for faults such as transverse cracks, inclined cracks, and clamp loosening in complex engine vibration spectra. This groundbreaking work demonstrates that existing aviation standard components can be transformed into intelligent nodes with self-sensing capabilities through minor structural modifications, providing new ideas for the lightweight design of future aviation equipment.

Going deeper into the engine, the main bearing, as the core component supporting the rotor, faces the most severe challenges of high temperature, oil mist, and thick metal casing shielding in its monitoring. The ultimate challenge in this field is how to achieve state monitoring in a “black box” environment without an external power supply and the inability to extract cables. Therefore, Zhang and his team developed a hybrid triboelectric variable reluctance generator (HTVRG) system (Fig. 9c) [217], creatively adopting a heterogeneous complementarity strategy to break this deadlock. In response to the weakness of TENG in driving high-power wireless transmission modules under high internal resistance, researchers have introduced a variable reluctance generator module to solve the energy consumption problem of wireless signals penetrating metal casings by utilizing its high power density characteristics. At the same time, utilizing TENG’s extremely high sensitivity to interface contact states, it accurately captures the slip characteristics of the bearing cage. The experiment shows that the system can complete wireless data transmission every 34 s even under low-speed conditions of 600 r min^−1^. This work demonstrates that TENG is fully capable of embedding itself as the core sensing unit in the heart of the engine, achieving wireless, passive real-time warning of deep component failures.

It is worth noting that the internal combustion chambers and turbine stages of aircraft engines generate temperatures that far exceed the thermal degradation limits of organic materials. Consequently, these triboelectric monitoring devices are typically deployed in relatively cooler peripheral regions rather than in the ultra-high-temperature core flow path. For instance, the aforementioned intelligent pipeline clamps are installed on external hydraulic lines outside the main casing, where temperatures are significantly lower. For internal components, such as accessory gearbox bearings, which still endure elevated temperatures and harsh oil-mist environments, researchers must employ specialized high-temperature-resistant engineering plastics. Advanced materials such as polyimide, polyetheretherketone, and specially aligned aramid nanofibers exhibit excellent thermal stability. These thermally stable polymers enable the sensors to maintain structural integrity and stable triboelectric charge density even at continuous operating temperatures approaching 300 °C. This strategic spatial deployment, combined with rigorous materials selection, ensures the practical viability of these sensors in harsh aviation environments.

As the construction of ultra-large space facilities expands toward in-orbit assembly, how to endow space robots with human-like fine touch to cope with blind operations in microgravity has become an urgent technological barrier to be broken through. Hou et al. designed a robot cluster system (S2A2RC) empowered by triboelectric sensors to address this challenge (Fig. 9d) [218]. The system integrates three types of sensors: TRLS (robotic leg), TRMS (robotic arm), and TSTS (truss), which, respectively, achieve precise closed-loop monitoring of crawling slip, grasping configuration, and mechanical locking status. The core breakthrough lies in using the metal truss body as a signal transmission medium, completely eliminating the hidden danger of cable entanglement. Experiments have shown that devices based on TC4 titanium alloy and PTFE exhibit excellent performance under vacuum and variable temperature (± 40 °C) conditions, providing key technical support for the autonomous maintenance of space-based defense systems.

Furthermore, Hou and his team developed a self-powered biomimetic whisker sensor (BMWS) inspired by mouse whiskers to address the recognition challenges of non-cooperative space targets such as space debris and failed satellites in backlit or blind spots (Fig. 9e) [219]. Unlike passive visual perception, BMWS utilizes active scanning of long whisker arrays to transform the three-dimensional geometric contours of an object's surface into distinct multi-channel triboelectric signals. Combining deep learning algorithms, BMWS has achieved precise classification of space debris such as solar cells in microgravity environments, providing a highly viable non-visual perception redundancy solution for future space robots to conduct surface morphology mapping of extraterrestrial celestial bodies in backlit areas and close-range reconnaissance of non-cooperative spacecraft.

After solving the problems of operation and perception, how to resist the impact of micro meteorites and space debris has become the key to the long-term survival of spacecraft. Traditional physical protective armor is often passive and imperceptible. Sun et al. combined metal 3D printing technology with TENG to prepare flexible and repairable metal fabrics with ball and socket interlocking structures (Fig. 9f) [220]. This smart armor not only retains the high-strength impact resistance of metal but also utilizes the triboelectric signals generated by impact to locate damaged areas in real time. The modular design, combined with spatial 3D printing technology, enables the rapid preparation and replacement of damaged units in orbit. This shift from one-time protection to renewable and perceptible protection greatly enhances the survival resilience of future space fortresses or deep space probes.

Going deep into the interior of the spacecraft, the flywheel system serves as the heart of attitude control, and its operational stability directly determines the pointing accuracy of the satellite. However, the extremely narrow non-guiding clearance inside the flywheel bearing has become a forbidden zone for traditional sensors. Gao and his team have designed an extremely compact embedded CL-TENG for this tiny space (Fig. 9g) [221]. They utilized ultra-thin flexible PCB technology and dielectric layer design to compress the thickness of the sensor to the extreme and successfully captured the micro-scale vortex and slip characteristics of the bearing cage without increasing mass or affecting the dynamic balance of the flywheel. This study provides a hidden online fault warning method for high-value military satellites, which can detect potential hazards in advance before attitude instability occurs.

For the monitoring of large strategic equipment, TENG’s micro-/nanotechnology advantages are reflected in its extremely high-voltage sensitivity and structural embeddability. Traditional active sensors are limited by cable layout and battery life, making it difficult to penetrate into narrow spaces such as engine interiors or flywheel clearances. TENG, utilizing the ultra-thin properties and self-powering mechanism of micro-/nanofilms, can be used as an intelligent pad implanted deep into mechanical structures. The response of the voltage signal generated based on the contact-separation mechanism to micro-scale structural damage is much higher than that of traditional vibration sensors, thus achieving on-demand maintenance for early failures of core components.

#### TENG-Enabled Devices for Planetary Exploration

Planetary exploration has become deeply intertwined with military strategy and competition for space resources. Mars and the moon are priority exploration targets, and their harsh environments (severe temperature differences, high radiation, and high-frequency dust storms) are not only ideal testing grounds for technological verification, but also directly related to the initiative of future deep space military deployment and resource control [222–226]. This type of detection task urgently requires durable and self-powered energy solutions, while traditional technology has obvious shortcomings: solar cells are prone to failure due to sand and dust obstruction, radioactive isotope thermoelectric generators pose pollution risks, and both are difficult to adapt to the core requirements of deep space exploration for lightweight and no external supply [227]. Planetary exploration missions such as Mars and the Moon require durable and self-powered energy solutions due to harsh environmental conditions such as severe temperature differences, high radiation, and high-frequency dust storms. TENG, with its outstanding advantages of strong material compatibility, flexible energy capture forms, and no need for external power sources, has gradually moved from laboratory verification to engineering applications. It can achieve invisible integration with detectors at low loads, achieving a breakthrough in the integration of power supply and perception. This feature significantly enhances the in-orbit operational resilience and detection accuracy of the probe, providing core technical support for the smooth advancement of deep space exploration missions, and has important strategic significance for seizing strategic high ground such as planetary exploration and space resources [228]. This section will review the research and application progress of TENG in planetary exploration.

**Fig. 10:**
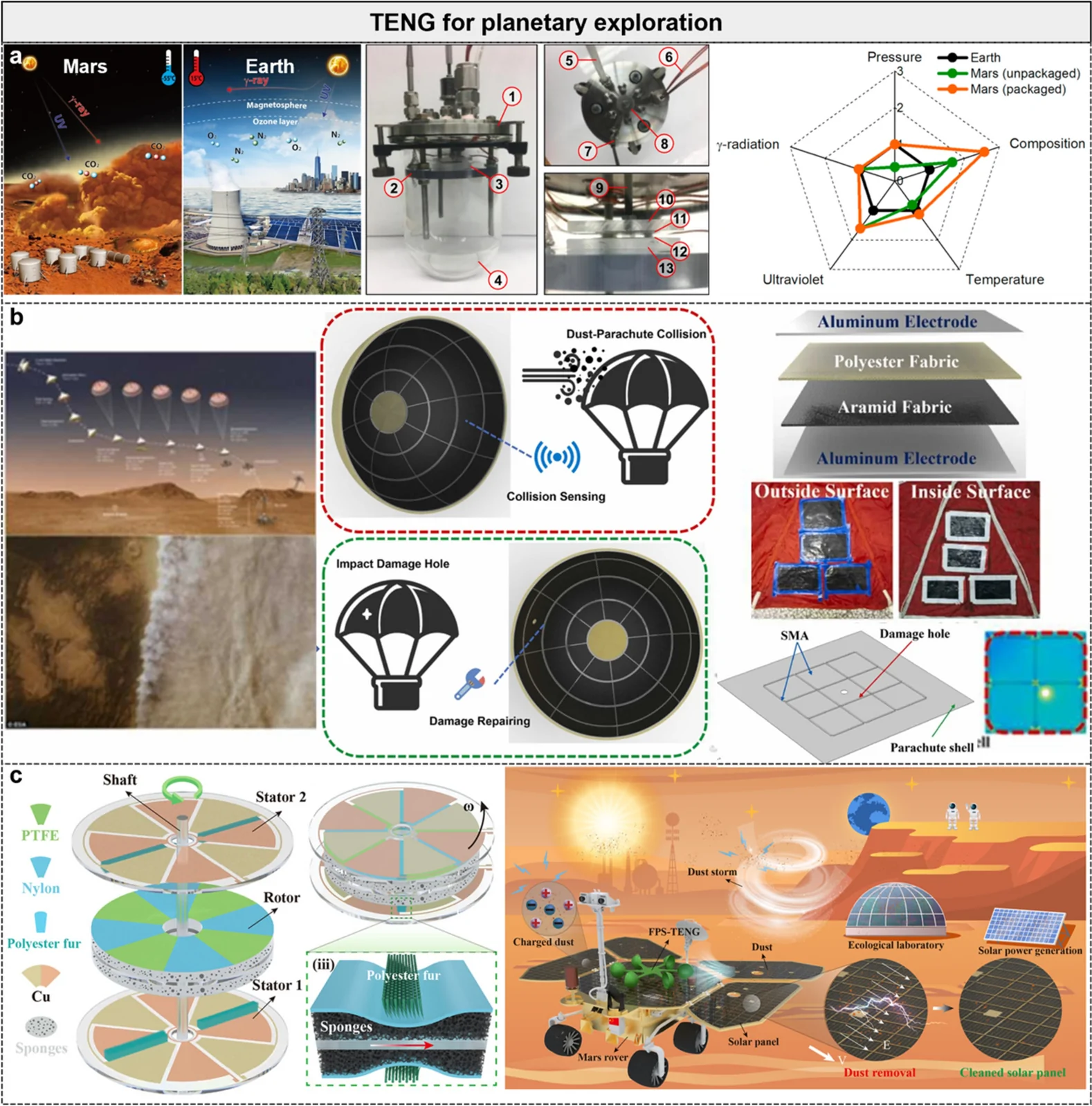
TENG for planetary exploration. **a** Environment and expected performance of Mars and Earth. Reproduced with permission from Ref. [229]. Copyright 2017, Elsevier. **b** Schematics of flexible self-perceiving/repairing parachute system. Reproduced with permission from Ref. [230]. Copyright 2022, Elsevier. **c** Schematic and application of FPS-TENG dust removal. Reproduced with permission from Ref. [231]. Copyright 2025, Wiley–VCH

At present, researchers have applied TENG to Mars exploration missions, and the primary bottleneck in this process is the continuity of energy supply. Traditional solar cells are susceptible to power outages caused by sandstorms, as well as the limitations of heavy and radioactive contamination risks associated with radioactive isotope thermoelectric generators. In response to this core issue, the Seol team’s research is not limited to simply verifying the power generation capacity of TENG. Instead, they have designed and utilized a Mars simulated climate module to achieve decoupling analysis of environmental factors, systematically studying the operating mechanism and performance of TENG in simulating extreme Martian environments (Fig. 10a) [229]. This study reveals that although the thin atmosphere on the surface of Mars can enhance the charge relaxation effect and reduce device performance by about 37%, the abundant carbon dioxide and strong ultraviolet radiation in the Martian atmosphere are actually favorable factors for improving triboelectrification performance. Its core value lies in breaking through the previous one-sided understanding that the Martian environment is not conducive to triboelectrification. Based on this discovery, the research team also proposed a strategy of using carbon dioxide gas-tight packaging, which can improve the output performance of TENG by 157% in the Martian environment compared to the Earth environment. This study not only demonstrates the feasibility of TENG as a lightweight auxiliary power source for Mars probes but also proposes an engineering concept for constructing a photovoltaic–triboelectric all-weather hybrid energy system, which has important guiding significance for the energy system design of future deep space exploration equipment.

On the basis of solving the continuity of energy supply, the equipment safety issues during the Mars Entry Descent Landing (EDL) phase have further promoted the evolution of TENG technology from single energy capture to multifunctional integration. This process often faces the threat of severe sandstorms, which makes the parachute fabric vulnerable to high-speed particle impact and damage, thereby endangering mission safety. Ding et al. designed and reported a flexible intelligent parachute system (FSPRP) that integrates TENG collision sensors triboelectric dust parachute collision sensors (TDPCS) and shape memory alloy (SMA) self-healing mechanism to address this pain point (Fig. 10b) [230]. By utilizing aramid and polyester fibers of the parachute body to construct a flexible sensor, it not only avoids additional load burden but also adapts to the lightweight requirements of the EDL stage. In addition, the sensor not only works stably in low-temperature and low-pressure environments on Mars, but also achieves precise damage quantification through a high linear relationship between output voltage and impact energy. This study not only overcomes the disadvantage of traditional rigid sensors being difficult to deploy on flexible parachutes but also provides a low-cost, lightweight, and highly reliable solution for intelligent perception and self-maintenance of future deep space exploration equipment in extreme environments.

With the advancement of Mars exploration from short-term roaming to long-term base construction, the long-term operation and maintenance of energy facilities have become a new bottleneck. The accumulation of dust on solar panels seriously weakens power supply efficiency, while traditional dust removal techniques require valuable electrical energy and are difficult to adapt to the Martian environment. Yang et al. proposed a targeted wind-driven ternary four-phase soft–soft contact TENG (FPS-TENG) [231], as shown in Fig. 10c. The ternary soft–soft contact structure of polyester brush sponge PTFE/nylon not only reduces device wear and improves durability, but also increases output voltage by 354% and 185%, respectively, compared to traditional binary/ternary TENG. More importantly, the device utilizes a special double-layer stacking structure to generate a four-phase AC high voltage, which excites a traveling wave electric field on the electrode surface, thereby driving the directional movement and removal of charged dust, overcoming the dependence of traditional standing wave dust removal on the inclination angle of the solar panel. The research results show that the system has excellent performance in simulating low-pressure and low-temperature environments on Mars, with a removal rate of up to 91.8% for simulated Martian dust without the need for an external power supply. This work provides a highly strategic solution for energy self-maintenance and dust prevention in long-term Mars exploration and base construction in the future.

Throughout the aforementioned research, the role of these devices in planetary exploration has evolved from that of a simple energy harvester to that of an intelligent microsystem core. From environmental adaptation and encapsulation to structural perception and self-driven active defense, this technological evolution clearly illustrates the development prospects of future deep-space equipment. It envisions a closed-loop ecosystem based on environmental energy utilization, self-perception, and self-maintenance. In future interstellar strategic competition, this self-powered technology, which breaks away from dependence on continuous Earth-based supply, may become a key variable. However, translating these promising simulation results into actual planetary missions requires careful consideration of spaceflight hardware qualification standards. To survive real mission conditions, these devices must demonstrate strong resilience against extreme temperature variations and long-term space radiation. Future engineering efforts must focus on selecting highly stable polymers and adopting reliable physical packaging to prevent charge dissipation in vacuum environments. Furthermore, these devices must undergo stringent vibration testing to ensure structural integrity during the high-energy launch phase. Fortunately, the inherent lightweight and solid-state architecture of these systems provides a strong foundation for meeting these survival criteria, while avoiding the severe leakage and explosion risks associated with traditional chemical batteries. Addressing these practical qualification challenges will be pivotal to certifying these self-powered systems for long-duration planetary surface missions.

### Special Tactical Scenarios

Beyond its large-scale applications on individual soldiers and main combat platforms, TENG is rapidly penetrating atypical combat domains such as weapon damage assessment and realistic training, thanks to its superior structural flexibility and wide range of material choices. This series of applications demonstrates the unique advantages of TENG technology in addressing specific battlefield challenges: namely, leveraging the sensitivity of micro-/nanostructures to overcome the physical limits of traditional sensors, and solving energy maintenance problems in extreme environments through self-powered characteristics.

**Fig. 11:**
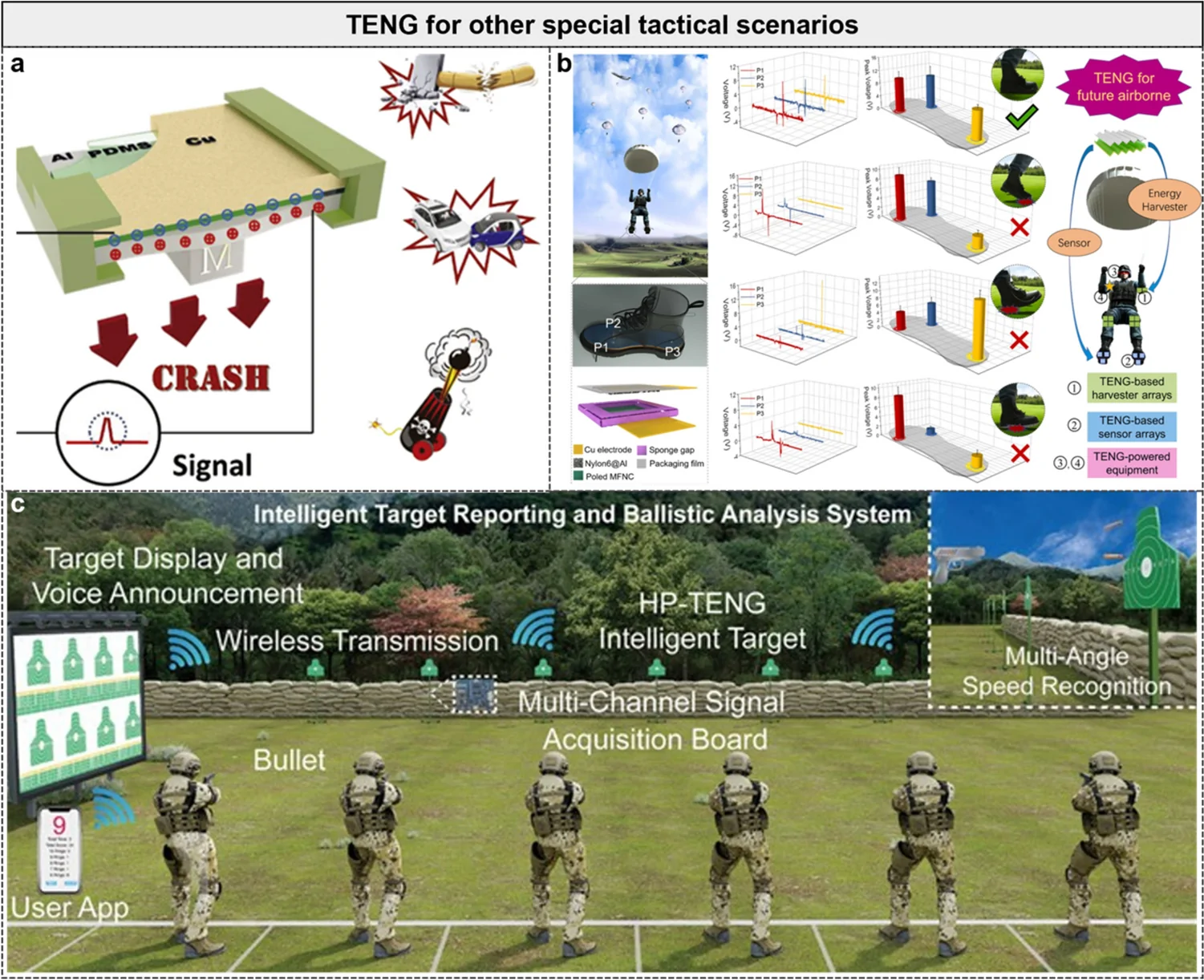
TENG for other applications. **a** Design of a self-powered accelerometer. Reproduced with permission from Ref. [232]. Copyright 2018, Elsevier. **b** Schematic diagram of the integration of wearable TENG devices for future airborne troops. Reproduced with permission from Ref. [233]. Copyright 2022, Elsevier. **c** A multifunctional impact sensing system integrating intelligent target reporting and ballistic analysis. Reproduced with permission from Ref. [234]. Copyright 2024, Wiley–VCH

In modern warfare, weapon systems (such as bunker buster bombs and penetrating weapons) and airborne personnel often need to withstand extremely high instantaneous overloads. Traditional active sensors often suffer data loss under such conditions due to battery failure or wiring breakage. Addressing the extreme monitoring requirements of gravitational accelerations (g) reaching tens of thousands of kilometers per second, Dai and his team pioneered the introduction of TENG technology into the field of MEMS microfabrication, designing a miniature, self-powered high-g acceleration sensor (Fig. 11a) [232]. This research eschewed complex piezoelectric stacking structures, instead employing a clever micro-spring-mass design to directly drive triboelectric signals through inertial impact. Its core breakthrough lies in demonstrating that the TENG maintains excellent linearity (*R*^2^ = 0.96) even under devastating impacts up to 1.8 × 10^4^ g. This provides a highly reliable passive black box solution for smart munitions, enabling precise recording of impact data at the moment of weapon penetration of cover, providing crucial support for damage assessment.

Simultaneously, for impact protection and monitoring of paratroopers landing, simple structural design is insufficient; material modification becomes critical. Chai et al., focusing on improving power density and charge stability, proposed a conductive sandwich-modulated ferroelectric nanocomposite material (AgNWs@PVDF-TrFE/BaTiO_3_) (Fig. 11b) [233]. This work effectively locks induced charges by introducing a conductive layer into the ferroelectric polymer to construct a deep-level potential well, breaking through the performance limits of single materials. The practical value of this technology lies in its demonstrated parachute landing monitoring function: by integrating high-performance materials into smart parachute boots, the system can quantify the impact force distribution and posture of soldiers upon landing in real time. This not only provides data support for preventing training injuries but also demonstrates the potential of TENG as a highly sensitive vibration sensor integrated into exoskeleton systems to achieve passive detection of micro-vibrations in the battlefield environment.

In the field of military training, traditional audio-visual target reporting systems suffer from high costs, difficult maintenance, and low recognition rates for multiple holes (multiple shots with the same hole). Sun’s research team constructed a flexible double-layer TENG array using high-temperature resistant polyimide, proposing a high-precision intelligent target range system (Fig. 11c) [234]. This system abandons expensive radar velocity measurement methods, utilizing the nanosecond-level signal time difference generated by bullets penetrating the double-layer film to accurately calculate the projectile's flight speed, impact coordinates, and incident angle. Its most disruptive contribution lies in overcoming the technical challenge of multiple hole recognition, achieving a 100% recognition rate at low cost. Looking to the future, this flexible sensor array could evolve into a bullet-sensing skin for individual body armor or armored vehicles, providing real-time feedback to the command center on the location of the hit and the extent of damage in actual combat, thereby achieving full-chain situational awareness from the training ground to the battlefield.

To systematically consolidate the complex interplay between specific tactical applications and their inherent engineering challenges, it is highly beneficial to cross-reference these parameters within a unified framework. Table 3 provides below systematically maps diverse military application domains to their dominant mechanical input types, recommended operational modes, and optimal material systems. More importantly, it explicitly aligns the primary technical bottlenecks identified in each domain with their corresponding state-of-the-art mitigation strategies. This macroscopic synthesis not only clarifies the structural and functional requirements tailored to specific battlefield environments but also serves as a practical engineering roadmap. By highlighting the critical transition from identifying environmental vulnerabilities to deploying targeted material- and circuit-level solutions, this table effectively bridges current technological capabilities and the future strategic priorities required for large-scale military deployment.

**Table 3:** Cross-reference of TENG applications in military fields, mechanical inputs, operational modes, materials, bottlenecks, and mitigation strategies

Platform	Application	Mechanical input	Mode	Materials	Bottleneck	Strategy
Individual soldier	Information security	Finger typing	Single-electrode	MXene/TPU, OML	Signal drift	Surface modification, adaptive baseline
	Wearable devices	Joint bending	Contact-separation	FAS-modified fabric, aramid aerogel	Sweat-induced charge loss	Superhydrophobic coating, sealed textile interface
	Tactical HMI	Tactile pressing	Single-electrode	Flexible hydrogels	Structural fatigue	Self-healing elastomer
Unmanned combat	Underwater operations	Water flow	Freestanding-layer	PVDF/MXene microfiber, elastomer/liquid metal	Signal attenuation, water interference	Bionic structure, hydrophobic interface
	Aerial vehicle	Aerodynamic flutter	Freestanding-layer	Lightweight polymer films	High cut-in speed, avionics mismatch	Bionic airfoil design, signal conditioning
Strategic equipment	Aerospace monitoring	Vibration, bearing rotation	Rolling	PI, PTFE, TC4 titanium, aramid nanofiber	Heat degradation	High-temperature polymers
	Planetary exploration	Dust impact	Contact-separation,	Aramid/polyester, polyester brush/PTFE	Dust accumulation	Electrostatic dust removal
Special tactical scenarios	Weapon assessment	Ballistic shock	Contact-separation	AgNWs@PVDF-TrFE/BaTiO_3_	Physical rupture	Spring-mass buffering

## Challenges and Strategies of TENGs in Military

**Fig. 12:**
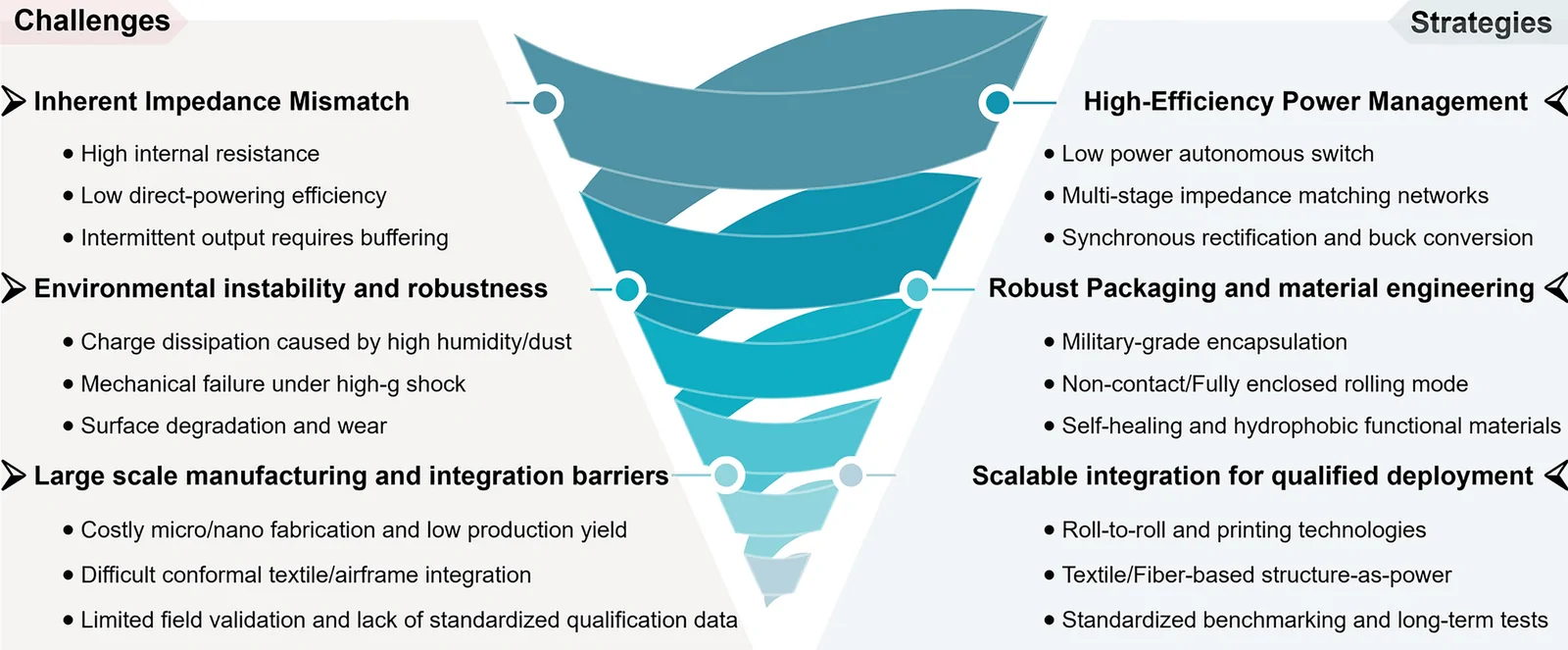
Challenges and strategies of TENG in the military field

Although TENGs have demonstrated remarkable high-voltage output and self-driving sensing capabilities in laboratory environments, transforming them into military equipment that meets practical requirements still faces a huge gap across basic physics, materials science, and systems engineering. The strict requirements for equipment in military operations, including extreme environmental tolerance, absolute reliability, and strict limitations on size, weight, and power consumption, pose unprecedented challenges to the engineering application of TENG. Therefore, it is not only necessary to recognize its inherent limitations as an emerging energy technology, but also to confront its engineering shortcomings exposed in complex battlefield environments. Figure 12 shows the challenges and strategies of TENG in the military field.

### Inherent Impedance Mismatch and Power Management Dilemmas

TENG is essentially a pulse AC power supply with high internal resistance (usually in the range of MΩ to GΩ), high voltage, and low current in physics. This output characteristic has a serious impedance mismatch with the majority of military standard electronic devices currently in use, which typically require low voltage, stable DC input, and low input impedance [235]. This fundamental difference leads to extremely low direct power supply efficiency, with the vast majority of energy dissipated in internal resistance. These inherent impedance mismatch and power management dilemmas manifest most critically in the practical applications discussed earlier in Sect. 3, particularly concerning individual wearable devices and unmanned combat systems. For instance, when attempting to power the distributed wireless sensor networks or radio-frequency communication modules integrated into an infantry soldier uniform, the high-voltage, low-current pulses generated by tactical biomechanical movements cannot directly drive these low-impedance standard military loads. The failure to effectively manage this precise energy conversion directly bottlenecks the critical transition of these wearable energy harvesters and unmanned aerial vehicle sensors from controlled experimental demonstrations to functional tactical gear deployed on the battlefield.

To systematically bridge the gap between abstract conceptual solutions and practical military applications, it is crucial to examine specific power management circuits designed for these devices and their demonstrated efficiencies. The core of solving this problem lies in developing dedicated and highly efficient power management integrated circuits that establish a material–circuit collaborative design paradigm [236]. Recent engineering advancements have successfully transitioned these strategies from theoretical models to highly efficient tangible hardware. For instance, the implementation of dedicated asynchronous high-voltage autonomous electronic switches paired with optimized buck converters has been proven to drastically improve energy transfer [237]. While the direct charging of low-impedance military loads from these high-resistance sources often yields extremely low power-transfer efficiency, state-of-the-art power management circuits utilizing synchronous rectification can remarkably boost this overall energy-conversion efficiency to a highly practical level. In actual military-relevant scenarios, such highly efficient circuits are indispensable. They can effectively convert the high-voltage intermittent alternating-current pulses generated by a soldier’s tactical movement or a vehicle’s mechanical vibration into a precisely regulated and steady direct current of typically 3.3 or 5 V. This standardized direct-current output has been successfully demonstrated to reliably power field-deployable military electronics, including distributed wireless sensor networks for battlefield environmental monitoring, infrared emitters for covert tactical signaling, and radio-frequency transmitters for zero-power communication [238]. Consequently, integrating these specific high-efficiency power management units, seamlessly combined with intermediate high-density supercapacitors to construct a multi-level impedance-matching network, not only functionally resolves the fundamental electrical mismatch but also decisively validates the feasibility of powering real-world autonomous nodes within the Internet of Military Things (IoMT) [239].

Furthermore, to fully resolve these power management dilemmas, it is imperative to shift from component-level concepts to a comprehensive system-level perspective. An illustrative end-to-end self-powered system architecture must seamlessly integrate the fundamental energy-harvesting interface with an impedance-matching power management unit, an intermediate energy buffer, and the final tactical payload. In realistic military scenarios, the power requirements for these payloads vary significantly. Distributed battlefield sensors, such as continuous temperature or acoustic monitors, typically operate within strict energy budgets in the microwatt to low-milliwatt range [240]. Conversely, active burst radio-frequency communication devices require transient power peaks of several tens of milliwatts to transmit encrypted data packets. Because the biomechanical or environmental energy inputs are inherently intermittent and produce high-voltage, low-current pulses, making it difficult to effectively drive these payloads directly, therefore, an intermediate buffering and storage approach is an absolute architectural requirement. The power management integrated circuit must effectively step down the voltage and transfer the extracted energy into high-density supercapacitors or solid-state micro-batteries to construct a multi-level impedance-matching network. Supercapacitors are particularly favored in tactical system architectures due to their rapid charge–discharge capabilities, theoretically infinite cycle life, and superior performance in extremely low-temperature environments where traditional chemical batteries routinely fail [241]. By establishing this complete end-to-end system architecture, engineers can precisely balance the intermittent energy generation rate with the time-averaged power consumption rate of the military sensors, thereby guaranteeing the continuous and reliable operation of autonomous tactical nodes.

### Stability and Robustness Crisis in Extreme Battlefield Environments

The battlefield environment is much harsher than controlled laboratories, covering high humidity, salt spray erosion, dust pollution, extreme temperature changes, and nuclear, biological, and chemical threats [242]. To bridge the immense gap between laboratory demonstrations and practical military deployment, the environmental tolerance of these functional devices must be rigorously quantified against stringent military specifications. Furthermore, in terms of complex mechanical robustness, devices integrated into tactical gear must endure complex random vibration profiles and survive functional shock impulses without experiencing physical fatigue, electrode detachment, or package rupture. In the specific context of hypersonic vehicle structural monitoring, extreme thermal shock and high-frequency aerodynamic flutter can rapidly induce polymer-matrix microcracking and irreversible electrode delamination. Similarly, for flexible wearable devices deployed in complex ground operations, continuous tactical movements combined with prolonged exposure to acidic sweat and abrasive desert dust inevitably lead to the physical abrasion of triboelectric interfaces and the severe structural fatigue of flexible conductive components.

**Fig. 13:**
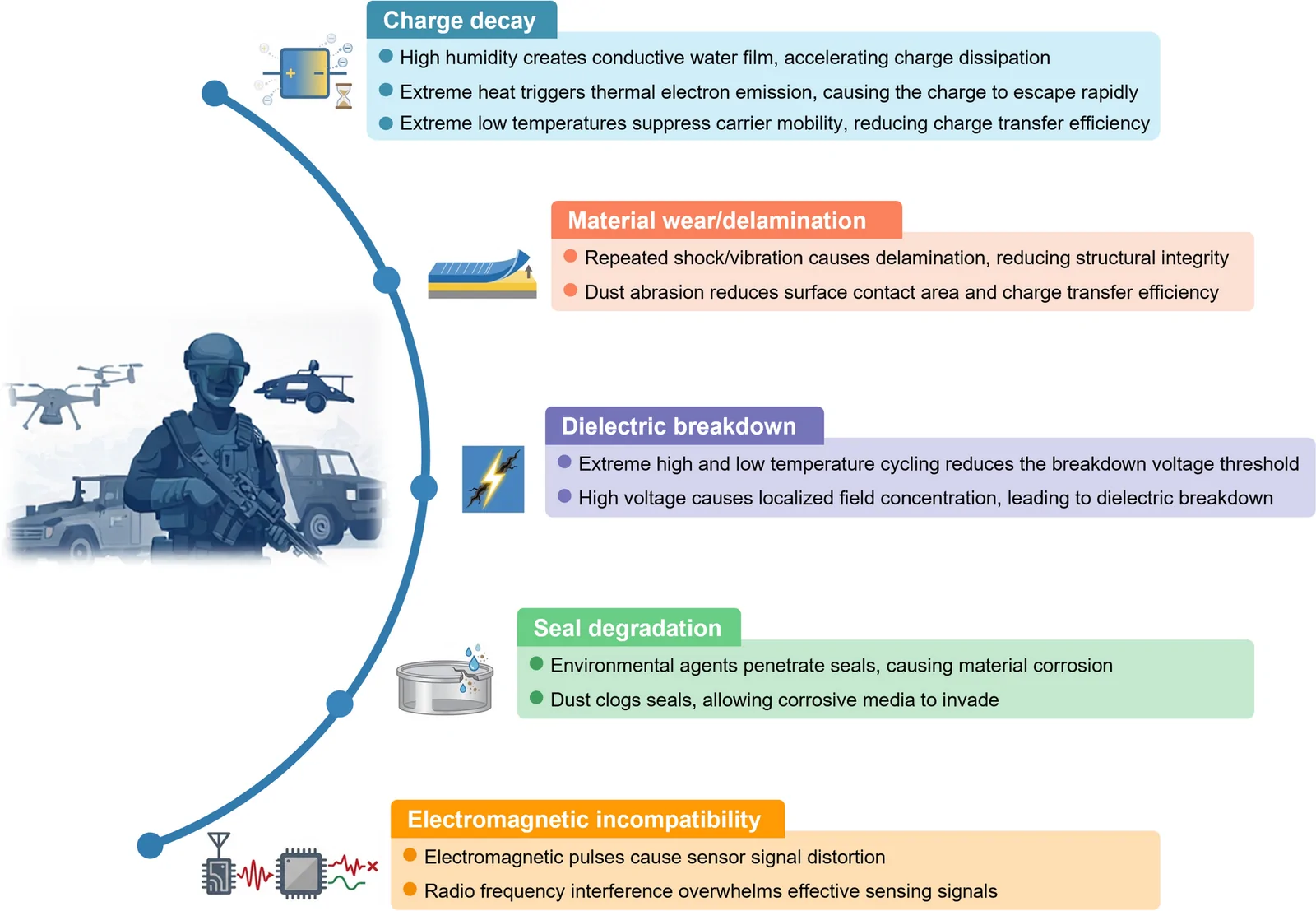
Integrated failure mode analysis under severe military stressors

Beyond immediate survivability against acute environmental shocks, long-duration military operations introduce severe reliability concerns regarding the continuous operational lifespan of these devices. In scenarios such as remote distributed sensor networks or extended planetary exploration, routine maintenance is structurally impossible. Over prolonged continuous operation, these energy harvesters face specific and unavoidable failure limitations that must be critically addressed before practical deployment. To systematically evaluate this long-term device reliability, it is essential to conduct an integrated failure-mode analysis detailing the underlying mechanisms and their direct correlations with specific environmental stressors, as visually summarized in Fig. 13. The primary degradation mechanisms span both physical and electrical domains and often interact synergistically to accelerate device failure. Charge decay represents a fundamental electrical failure mode, in which high ambient humidity creates a conductive surface water film that accelerates charge dissipation, while extreme heat triggers thermal electron emission and extreme cold severely suppresses internal carrier mobility [243]. Mechanically, continuous operation relying on interfacial friction inevitably induces material wear, physically destroying the delicate surface microstructures engineered for optimal contact area and resulting in a steady and irreversible decline in electrical output [244]. Furthermore, repeated ballistic mechanical shocks and extreme vacuum thermal cycling frequently lead to structural delamination, in which severe thermal stress gradients cause irreversible interfacial separation between the conductive electrodes and the dielectric polymers [245]. Electrically, the inherent high-voltage operation introduces a severe risk of dielectric breakdown, particularly in aggressively thinned wearable applications, resulting in catastrophic permanent short circuits [246]. These internal component failures are frequently exacerbated by overall packaging seal failure, in which prolonged chemical exposure and temperature fluctuations degrade the encapsulant, allowing the rapid ingress of corrosive moisture and abrasive dust to directly block the contact electrification process [247]. Finally, regarding overall system integration, electromagnetic compatibility concerns constitute a critical failure mode because the high-voltage pulsed outputs can generate broad-spectrum electromagnetic interference, potentially disrupting adjacent highly sensitive military avionics or revealing the user to enemy electronic reconnaissance [248]. To mitigate these critical long-term failure modes and ensure absolute reliability in maintenance-free military operations, future engineering efforts must adopt a comprehensive strategy of both internal material optimization and external structural protection. At the fundamental material level, the development of modified, highly stable polymer dielectrics that are essentially superhydrophobic, antistatic, and possess intrinsic self-healing functions forms the foundational requirement [249]. Concurrently, at the macroscopic structural level, it is absolutely necessary to introduce aerospace-grade hermetic packaging technologies and transition device designs from single contact-separation modes to fully enclosed non-contact or rolling modes, so as to physically isolate the delicate charge-generation interfaces from the interference of external environmental contaminants [250, 251]. Synthesizing these interconnected failure mechanisms provides a comprehensive and objective engineering foundation for developing the targeted structural mitigation strategies required for actual combat deployment.

### Engineering Barriers to Scalable Manufacturing and Seamless Integration

Currently, high-performance TENG devices rely heavily on laboratory-level micro-/nano-processing techniques such as photolithography and plasma etching, which are costly and difficult to achieve large-scale, continuous industrial production [252]. More importantly, military equipment requires that the energy system must have a high degree of integration and concealment, without adding extra weight to soldiers or changing the aerodynamic shape of the equipment [253]. These engineering barriers to scalable manufacturing and seamless integration are the primary real-world obstacles preventing the widespread tactical deployment of the critical applications discussed previously in Sect. 3. For example, wearable devices and human–machine interaction systems inherently require large-scale roll-to-roll manufacturing of textile-based energy harvesters to become economically viable for equipping entire infantry divisions with intelligent combat uniforms. If these highly flexible sensors cannot be seamlessly integrated into the structural fabric at the foundational industrial manufacturing stage, they will inevitably add prohibitive parasitic weight and severely restrict the physical mobility of combat personnel. Simultaneously, for unmanned aerial vehicles and aviation equipment monitoring applications, the profound difficulty in achieving conformal structural integration without altering the aerodynamic profile directly limits their practical utility, because rigid and bulky forms would significantly increase aerodynamic drag and radar cross sections, thereby compromising essential stealth operations.

Moving beyond the theoretical feasibility of roll-to-roll processing and textile integration, it is critical to evaluate the specific micro- and nanostructuring techniques most viable for actual industrial production. While laboratory-scale devices often rely on extreme-precision techniques like electron-beam lithography or deep reactive-ion etching to maximize surface area, these batch processes are economically and temporally prohibitive for mass manufacturing [254]. For large-scale military deployment, continuous nanoimprint lithography and rapid laser texturing emerge as the most viable and scalable production techniques [255, 256]. However, scaling these physical processes introduces severe challenges regarding manufacturing tolerances and production yields. Maintaining strict nanoscale uniformity over extensive flexible substrates is highly susceptible to local mechanical defects and particle contamination, which drastically reduces the overall yield of continuous production lines. To achieve realistic industrial yields, engineering efforts must shift toward optimizing hierarchical microscale structures, where relaxed manufacturing tolerances do not catastrophically compromise triboelectric surface charge density [257]. Furthermore, the primary cost drivers in current fabrication paradigms are the reliance on expensive consumable master molds and energy-intensive vacuum-deposition processes. Achieving cost-effective mass production for military procurement will mandate a decisive transition toward reusable metallic or robust polymeric texturing rollers, combined with the widespread adoption of ambient solution-based coating techniques [258].

Therefore, overcoming these bottlenecks is an absolute prerequisite for translating these self-powered applications from bespoke laboratory prototypes into mass-produced military assets. Breaking through the manufacturing bottleneck necessitates heavily drawing upon the mature experience of printed flexible electronics and advanced textile engineering [259, 260]. The rapid development of low-cost manufacturing technologies based on continuous roll-to-roll processing, utilizing the aforementioned reusable texturing rollers and ambient coating techniques, is a mandatory prerequisite for achieving large-scale defense deployment [261]. Furthermore, in terms of tactical structural integration, the engineering focus must strictly remain on developing continuous fiber-level and fabric-level triboelectric generators, employing mature industrial textile technologies to directly weave functional conductive fibers into the inner lining of combat suits, thereby truly achieving the seamless integration of protective structure and autonomous power generation [262, 263]. By systematically bridging these identified material, circuit, and manufacturing bottlenecks, the scientific community can establish a highly transparent and actionable engineering roadmap, accelerating the ultimate transition of these innovative self-powered systems into battle-ready defense assets.

Traditional energy storage solutions such as lithium-ion batteries currently dominate military logistics due to their exceptionally high continuous energy density [264]. However, these chemical batteries are fundamentally limited by finite operational lifespans, heavy logistical resupply requirements, and severe safety vulnerabilities regarding thermal runaway upon ballistic penetration [265, 266]. In contrast, while TENGs currently exhibit significantly lower absolute energy density, they offer near-infinite operational lifespans, zero-maintenance logistical burdens, and intrinsic material safety under extreme mechanical shock. Furthermore, when evaluated as autonomous sensors and compared against mature piezoelectric ceramics, these devices demonstrate vastly superior sensitivity to low-frequency, disordered mechanical stimuli and offer unparalleled structural flexibility for conformal wearable integration. Nevertheless, piezoceramics still maintain a distinct advantage in high-frequency resonance and absolute charge retention under high ambient humidity [267]. To provide a rigorous engineering perspective for future military procurement, the newly added Table 4 systematically consolidates these key metrics, clarifying the distinct tactical niches in which self-powered materials can effectively augment or eventually replace conventional military electronics.

**Table 4:** Quantitative comparison of TENG with metrics of other military technologies

Technology	Energy density	Operational lifespan	Size and weight	Environmental adaptability
Lithium-ion batteries	150–250 Wh kg^−1^	500–1000 standard charge cycles	Bulky and heavy	− 45 to 70 °C, severe thermal runaway risk under impact
Piezoelectric sensors	mW cm^−3^ level, resonant output	Commonly 10^5^–10^7^ cycles, but ceramic systems remain vulnerable to brittle fatigue and bonding degradation	Ceramic PZT is dense and mostly rigid, about 7.5 g cm⁻^3^	− 45 to 200 °C, poor low-frequency response
Triboelectric nanogenerators	mW cm^−3^ under defined excitation	Commonly > 10^6^ cycles without obvious degradation	Polymer- and textile-based, often < 2 g cm⁻^3^	− 45 to 150 °C, output degrades under high humidity

While this theoretical benchmarking highlights the distinct tactical advantages of triboelectric systems, verifying these capabilities requires moving beyond controlled conditions. Beyond specific material and circuit-level bottlenecks, evaluating the critical transition from laboratory environments to real-world field validation is absolutely essential for assessing actual military adoption. It is important to note that triboelectric devices have already demonstrated significant resilience in simulated extreme environments, including continuous exposure to high temperatures, elevated ambient humidity, and extreme cold [268–270]. More importantly, preliminary field validations have been successfully executed in unscripted harsh scenarios such as actual marine environments and polar regions [271–275]. These successful real-world deployments effectively prove the fundamental survivability of these self-powered materials and strongly validate their readiness to guide practical tactical applications. However, to fully meet rigorous military qualification standards, the current scope of field testing must be substantially expanded. The existing literature regarding actual field deployment is currently limited by small testing sample sizes, relatively short evaluation durations, and a notable scarcity of combined testing under multiple synergistic environmental stressors. Addressing these specific developmental gaps is an urgent requirement for the scientific community. Future research must prioritize extensive and long-term field deployment under coupled multi-environmental conditions to decisively elevate the overall technological feasibility and accelerate the ultimate transition of these self-powered systems into battle-ready defense assets.

## Conclusions and Future Prospects

Since their inception, TENGs have triggered a paradigm shift in the fields of distributed energy harvesting and self-powered sensing, owing to their unique contact electrification mechanism and broad material versatility. As a potentially disruptive technology, TENGs, with their inherent advantages of self-powering, high concealment, and flexible integration, provide a new technological route for overcoming the bottlenecks of energy endurance and intelligent perception in modern military equipment. This review systematically summarizes the latest research advances and application progress of TENGs across four overarching military platform categories. Specifically, it examines the integration of biomechanical energy harvesting and tactical interfaces in individual soldier combat platforms, the environmental energy harvesting and covert perception capabilities of unmanned combat platforms, the auxiliary power autonomy and passive structural monitoring of strategic equipment platforms, and the survivability of TENG-enabled systems under extreme conditions in special tactical scenarios. It also analyzes the core challenges involved in the transition from laboratory prototypes to practical military deployment, including energy output density, environmental adaptability, and system-level compatibility. However, it must also be recognized that current military research in this field remains at a critical stage in the transition from basic science to engineering application. Although laboratory results are often encouraging, they still fall short of the rigorous requirements for practical deployment, particularly in terms of dynamic impedance matching, long-term robustness in extreme environments, and seamless compatibility with established military standards.

**Fig. 14:**
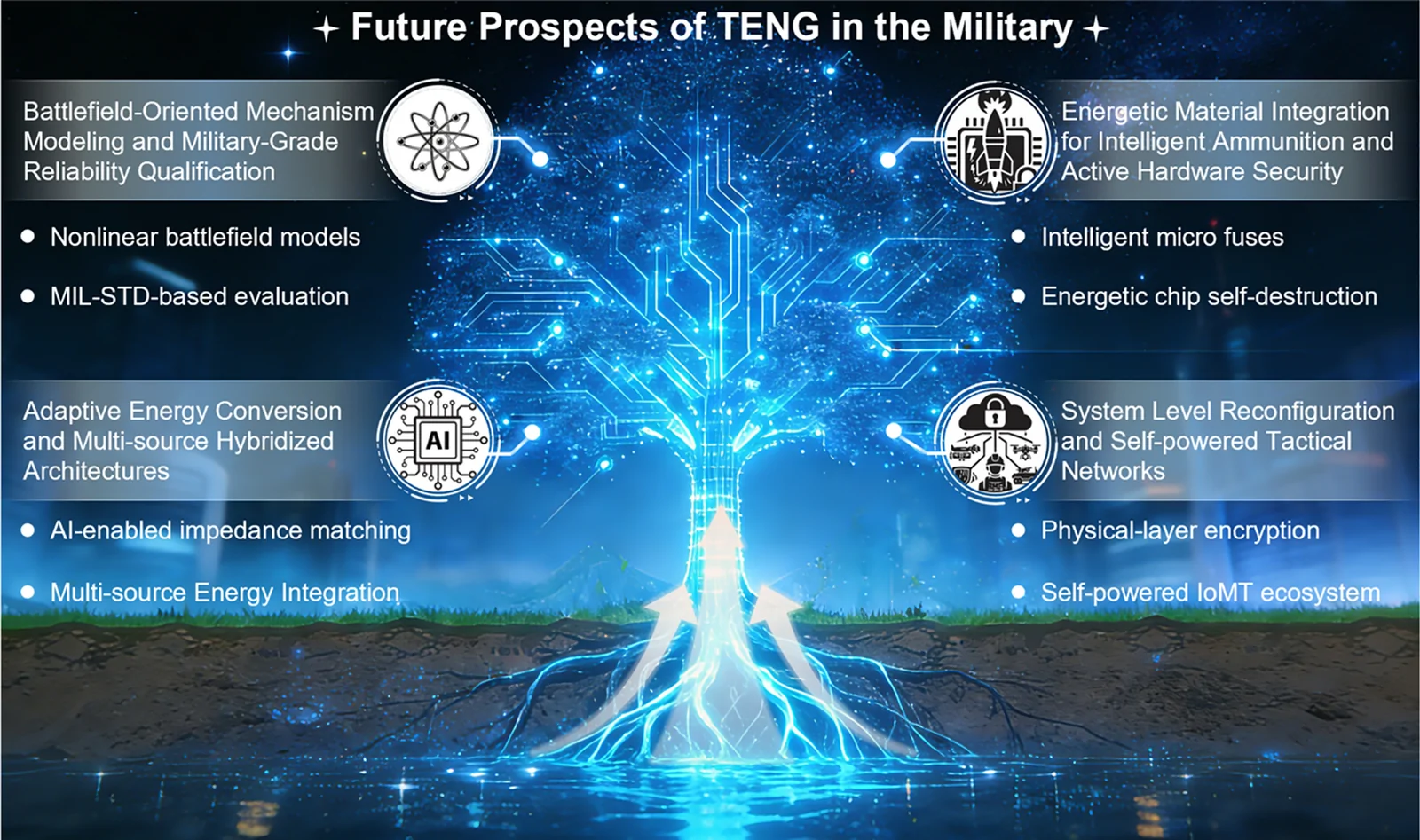
Future prospects of TENG in the military field

To fully realize the strategic dominance of these self-powered systems in future warfare, research must pivot from laboratory proof of concept demonstrations to deep system-level integration capable of withstanding the uncompromising rigors of the battlefield across all aforementioned platform scales. Consequently, this review has synthesized the highly diverse forward-looking directions into four overarching strategic priorities (Fig. 14).

### Battlefield-Oriented Mechanism Modeling and Military-Grade Reliability Qualification

As the primary strategic priority, future theoretical research must transcend ideal laboratory conditions to establish nonlinear dynamic models applicable to high-entropy mechanical energy, introducing surface-wear attenuation factors and force–electric coupling terms to correct displacement current equations under extreme dynamic loads such as explosive shocks and chaotic tactical movements. Simultaneously, it is imperative to establish a concise testing framework strictly aligned with rigorous military-grade stressors. To systematically guide future technological development, we have proposed a comprehensive testing framework and a minimal set of reporting standards detailed in Table 5. Instead of conducting generic laboratory evaluations, future research must engage deeply with specific military parameters and subject these devices to an evaluation matrix strictly benchmarked against established guidelines such as MIL-STD-810H for environmental testing, MIL-STD-461G for electromagnetic compatibility, and MIL-STD-883 for ionizing radiation. For example, researchers must critically evaluate device survivability under condensing humidity and corrosive salt spray, referencing Methods 507 and 509, assess structural integrity against complex random vibration profiles under Method 514, and track frictional surface wear under high-velocity abrasive dust testing based on Method 510. Concurrently, to ensure data comparability and reproducibility across the global research community, researchers should be systematically required to uniformly report baseline charge density and power output metrics both before and after exposure to these specific environmental stressors. Furthermore, experimental studies must document continuous performance degradation curves over standardized environmental or mechanical cycles rather than merely reporting peak initial performance values. For specialized applications involving electronic warfare or space deployment, reporting standardized electromagnetic shielding effectiveness and quantifiable radiation tolerance thresholds must become mandatory. To guarantee absolute reliability under these standardized stresses, future efforts must develop advanced molecular-level self-healing polymer architectures and airtight packaging strategies capable of recovering electrical properties after devastating physical impacts. To truly overcome these operational bottlenecks, future investigations must deeply address concrete research questions, such as quantitatively decoupling the synergistic effects of continuous mechanical wear and extreme temperature cycling on the interfacial charge transfer mechanism to mathematically predict the absolute operational lifespan of these devices in harsh battlefield environments.

**Table 5:** Concise testing framework integrating specific military standard parameters

Military-grade stressor	Test parameters	Reporting standard
Humidity	Condensing humidity exposure benchmarked under MIL-STD-810H method 507	Power degradation curves, charge density retention, and optical moisture ingress verification
Dust	High velocity abrasive fine dust under MIL-STD-810H method 510	Frictional surface wear quantification and continuous electrical output tracking
Salt spray	Prolonged corrosive salt spray cycles under MIL-STD-810H method 509	Electrode physical degradation tracking, and continuous electrical output stability after exposure
Thermal cycling	Shock from constant extreme temperatures under MIL-STD-810H method 503	Material structural integrity observations, and continuous electrical output stability across the temperature spectrum
Vibration	Complex random vibration profiles under MIL-STD-810H method 514	Output voltage fluctuations during mechanical loads, and specific physical delamination rates
Electromagnetic interference	Radiated and conducted emissions testing strictly under MIL-STD-461G	Standardized electromagnetic shielding effectiveness and precise radiated emission levels
Ionizing radiation	Prolonged ionizing dose exposure testing under MIL-STD-883 method 1019	Quantifiable radiation tolerance thresholds and corresponding dielectric breakdown limits

### Adaptive Energy Conversion and Multi-source Hybridized Architectures

As the second strategic priority, the research focus must address the high degree of randomness in battlefield energy sources, which, combined with the inherently high internal resistance of these devices, results in serious impedance mismatches. To fundamentally resolve these power management dilemmas, future engineering efforts must shift toward artificial-intelligence-enhanced power management chips capable of instantly analyzing the chaotic frequency characteristics of unscripted battlefield stimuli and dynamically adjusting circuit impedance to maximize real-time energy extraction. This proactive energy management strategy, combined with the heterogeneous integration of diverse energy sources such as photovoltaic and thermoelectric systems, will decisively fill the energy gap in low-frequency and low-light environments, thereby forging highly resilient and all-weather battlefield energy networks. Realizing this proactive network intrinsically depends on solving specific research questions, particularly how ultra-low-power machine learning algorithms can be embedded directly onto tactical sensor nodes to achieve instantaneous impedance matching during unpredictable combat maneuvers without draining the harvested energy buffer.

### Energetic Material Integration for Intelligent Ammunition and Active Hardware Security

The third strategic priority focuses on the deep integration of these self-powered devices with various energetic materials to revolutionize intelligent ammunition and active hardware security. Traditional weapon fuses and data destruction mechanisms heavily rely on chemical batteries and complex backend circuits, which occupy valuable internal space and severely limit long-term operational lifespans. Because these devices can naturally produce extremely high open-circuit voltages upon instantaneous mechanical impact, they present a highly practical opportunity to directly drive microelectromechanical fuses and ignite pyrotechnic materials for intelligent munitions. Furthermore, beyond direct high-voltage ignition, the ultrahigh sensitivity of these functional polymers to external mechanical stimuli offers a highly concealed trigger mechanism for critical information security. Future research should strategically explore coupling these self-powered sensory arrays with micro-energetic payloads integrated directly onto critical military semiconductor chips. Upon unauthorized physical tampering or vehicle capture, these integrated sensory skins can instantly detect anomalous mechanical intrusion and reliably trigger the energetic materials to achieve complete physical hardware self-destruction, thereby ensuring absolute data security. This versatile integration strategy, combining both direct high-voltage detonation and sensitive mechanical triggering, opens a highly critical research avenue where future studies must address the concrete question of optimizing interfacial material designs to ensure that these electrostatic discharges reliably detonate insensitive munitions or self-destruct payloads without accidental misfires during high-gravity launch phases or intense battlefield vibrations.

### System-Level Reconfiguration and Self-powered Tactical Networks

The ultimate strategic priority is elevating these autonomous nodes to construct a comprehensive and self-powered global military Internet of Things ecosystem. Currently, battlefield situational awareness is severely constrained by the limited battery life of distributed sensors and the logistical impossibility of replacing thousands of batteries during active combat operations. By integrating maintenance-free functional sensors into all physical assets, ranging from individual infantry gear to large-scale strategic vehicles, the military can achieve unprecedented high-density ubiquitous sensing. Furthermore, the unique electrical signals generated by these devices can be directly utilized for hardware-based physical layer encryption, providing a secure communication barrier that is highly resistant to traditional software cyber-attacks. By completely eliminating the fundamental dependence on traditional power grids and vulnerable chemical batteries, this comprehensive integration will establish a truly decentralized and highly concealed sensing network, successfully achieving the strategic goal of real-time global perception in modern warfare. However, translating this conceptual network into actual combat capability requires researchers to deeply address specific tactical questions, such as mathematically transforming the unique triboelectric signal fingerprints generated by infantry movements into dynamic cryptographic keys that strictly meet military-grade encryption standards for secure swarm drone communication.
